# Protein Kinase Serine/Threonine Kinase 24 Positively Regulates Interleukin 17-Induced Inflammation by Promoting IKK Complex Activation

**DOI:** 10.3389/fimmu.2018.00921

**Published:** 2018-04-30

**Authors:** Yu Jiang, Miao Tian, Wenlong Lin, Xinyuan Wang, Xiaojian Wang

**Affiliations:** ^1^Department of Clinical Laboratory Medicine, Second Affiliated Hospital, School of Medicine, Zhejiang University, Hangzhou, China; ^2^School of Medicine, Institute of Immunology, Zhejiang University, Hangzhou, China

**Keywords:** serine/threonine kinase 24, experimental autoimmune encephalomyelitis, interleukin 17, IKK, inflammation

## Abstract

Interleukin 17 (IL-17) is a key inflammatory cytokine that plays a critical role in tissue inflammation and autoimmune diseases. However, its signaling remains poorly understood. In this study, we identified serine/threonine kinase 24 (Stk24) as a positive modulator of IL-17-mediated signaling and inflammation. Stk24 deficiency or knockdown markedly inhibited IL-17-induced phosphorylation of NF-κB and impaired IL-17-induced chemokines and cytokines expression. Stk24 overexpression greatly enhanced IL-17-induced NF-κB activation and expression of chemokines and cytokines in a kinase activity-independent manner. The IL-17-induced inflammatory response was significantly reduced in Stk24-deficient mice. In addition, the severity of experimental autoimmune encephalomyelitis was markedly reduced in mice with a deficiency of Stk24 in non-hematopoietic cells. We further demonstrated that Stk24 directly interacts with TAK1 and IKKβ and promotes the formation of TAK1/IKK complexes, leading to enhanced IKKβ/NF-κB activation and downstream cytokines and chemokines induction. Collectively, our findings suggest that Stk24 plays an important role in controlling IL-17-triggered inflammation and autoimmune diseases and provides new insight into the therapeutic targets of IL-17-mediated inflammatory disease.

## Introduction

The interleukin 17 (IL-17) family is a recently emerged subset of cytokines secreted by innate and adaptive immune cells, including iNKT cells, δγ T cells, LTi-like cells, NK cells, and myeloid cells, and participate in both acute and chronic inflammatory responses (1). This family contains six members: IL-17A, IL-17B, IL-17C, IL-17 D, IL-17E (also named IL-25), and IL-17F. IL-17A (commonly referred to as IL-17), a founding member of the IL-17 family, is the most widely investigated cytokine of this family and its biological function and regulation are best understood. IL-17F shares high homology with IL-17A and resembles IL-17A in both its cellular sources and regulatory functions (2, 3). IL-17 acts on multiple cell types, including endothelial cells, fibroblasts, epithelial cells, and astrocytes, and coordinates local tissue inflammation by promoting the generation of pro-inflammatory and neutrophil-mobilizing cytokines and chemokines (4, 5). IL-17 has been linked to the pathogenesis of autoimmune diseases, including multiple sclerosis (MS). IL-17 levels are increased in patients with MS (6). Moreover, studies using an animal model of MS [experimental autoimmune encephalomyelitis (EAE)] have showed that IL-17 is responsible for disease development (7).

Interleukin 17 family receptors, which consist of IL-17RA, IL-17RB, IL-17RC, IL-17RD, and IL-17RE, contain a fibronectin III-like domain in their extracellular region and an SEF/IL-17R (SEFIR) domain in their intracellular region. IL-17R signaling transduces *via* a heteromeric receptor complex composed of two receptor chains: IL-17RA and IL17-RC (2, 8). Adaptor protein NF-κB factor 1 (Act1) is recruited to the receptor complex *via* homotypic interactions of the SEFIR domains upon IL-17 stimulation (9). In turn, Act1 recruits TRAF6 and mediates the Lys63 ubiquitination of TRAF6 (10, 11). Poly-ubiquitinated TRAF6 further activates TAK1, which mediates the activation of NF-κB, MAPK cascades, and the transcription factor C/EBP, resulting in the upregulated expression of various pro-inflammatory chemokines and cytokines and subsequent myeloid cell recruitment to inflamed tissue (12). In addition to the activation of TRAF6, Act1, which is phosphorylated by IKKi, also recruits TRAF2 and TRAF5 to prolong the half-life of IL-17-stimulated mRNA (13, 14). Recently, more and more molecules were shown to participate in IL-17R signaling. USP25 directly removes the IL-17-induced ubiquitination of TRAF5 and TRAF6 and consequently suppresses both NF-κB activation and mRNA stabilization pathways (15). TPL2 binds to TAK1 and mediates the phosphorylation and catalytic activity of TAK1, thereby promoting IL-17-stimulated gene expression (16). TRAF3 binds to IL-17R directly and interferes with the IL-17R–Act1–TRAF6 complex, thus restraining the activity of IL-17R signaling (10). We recently identified that nuclear Dbf2-related kinase 1 (NDR1, stk38) functions as a positive regulator of IL-17R signal transduction and IL-17-induced inflammation by competitively binding to TRAF3 (17). However, details on other molecular functions regarding the regulation of downstream pathways linked to IL-17R signaling remain unclear.

Serine/threonine kinase 24 (Stk24), also known as mammalian STE20-like kinase 3, belongs to the Ste20 serine/threonine protein kinase family and subfamily of germinal center kinase III kinases (18, 19) and is involved in a wide range of basic cellular processes. Stk24 positively regulates the cell cycle, cell growth, migration, and synapse development in a kinase activity-dependent manner (20–23). In addition, Stk24 promotes proliferation and tumorigenicity *via* the VAV2/Rac1 signaling axis as an adapter (24). Using Stk24^h/h^ mouse in which the Stk24 gene was disrupted by an insertion, Zhang et al. have demonstrated that the interaction complex of Stk24 and CCM3 as being an important regulator of neutrophil degranulation by coordinating UNC13D-driven vesicle exocytosis in neutrophils (19). However, the function and signal transduction of Stk24 in inflammation is poorly illustrated.

Here, we report that Stk24 positively modulates IL-17-mediated signaling and inflammation. Stk24 promotes IL-17-triggered phosphorylation of the NF-κB P65 subunit and induction of pro-inflammatory cytokines and chemokines in a kinase-independent manner. Stk24-deficient mice are resistant to IL-17-mediated inflammation and MOG-induced EAE. An additional study showed that Stk24 directly binds with TAK1 and IKKβ and promotes the formation of the TAK1/IKK complex, resulting in enhanced activation of the IKKβ/NF-κB pathway and downstream inflammatory factor production.

## Materials and Methods

### Reagents and Antibodies

Recombinant IL-17A (mouse and human) were purchased from PeproTech (PeproTech, Rocky Hill, NJ, USA); human IL-17F (11855-HNAE), IL-1β, and TNF-α were purchased from Sino Biological Inc.; Primary antibodies against P-P65 (3033S), P-P38 (4511S), P-ERK1/2 (4370S), P-JNK (9251S), P-IκBα (2859), P-IKKα/β (2697), P-TAK1 (4508), IκBα (4814), P65 (8242), P38 (8690), and ERK1/2 (4695) were purchased from Cell Signaling Technology. Primary antibodies for Stk24 were purchased from Abcam (ab155198), IKKα/β (sc-7607), IKKγ (sc-8330), and mouse control IgG were purchased from Santa Cruz Biotechnology. Anti-Flag (M2) beads were purchased from Sigma. Antibodies against Flag, HA, Myc, and anti-HA beads were purchased from Abmart. Antibody for GADPH (M130718) was purchased from Huabio. PEI was purchased from Polyscience. INTERFERin@ was purchased from Polyplus Transfection.

### Mice

Stk24^h/+^ mice were kindly provided from Dr. Dianqing Wu in Yale University and maintained in the specific pathogen free facility of The University of Zhejiang. The Stk24^h/+^ mice were crossed to get age- and sex-matched wild-type (WT) or Stk24^h/h^ littermates. Mice were genotyped by PCR analysis of isolated tail DNA using the following primers as pervious reported (19): 5′-AAAGCGGTGGGGAAATTAGAAAA-3′; 5′-CTCTGTATAGCCCTGGCTGCATACAA-3′; 5′-GGCACCCACGACCTGGCTTA-3′. All animal experiments were performed in accordance with protocols approved by the Institutional Animal Care and Scientific Investigation Board of Zhejiang University (Authorized N.O. ZJU2015-040-01).

### Plasmids

Serine/threonine kinase 24, TRAF6, IKKα, IKKβ, and IKKγ were amplified from HeLa cells by PCR and ligated into pIRES-Flag/HA to construct Flag- or HA-tagged expression plasmids. The pCMV6-XL5 vector, expressing human full-length and truncated Stk24, was amplified from HeLa cells by PCR. Stk24 was also ligated into pMXS-IRES-EGFP for the retrovirus-mediated restoration of gene expression in mouse embryo fibroblasts (MEFs).

### Retrovirus-Delivered Gene Overexpression

Plate E cells were transfected with pMXS-IRES-EGFP-Flag-Stk24 or pMXS-IRES-EGFP using the calcium phosphate transfection method for viral packing, and the supernatant was harvested to obtain virus. WT or Stk24^h/h^ MEFs were infected with viral supernatant, after 2 days, cells were stimulated with IL-17A for the indicated times before real-time PCR and immunoblot analysis.

### Cell Culture, Plasmid Transfection, and Small Interfering RNA (siRNA) Silencing

HeLa cells, human HEK293T cells, plate E cells, WT, or Stk24^h/h^ MEFs were cultured in DMEM supplemented with 10% FBS. Primary MEFs were isolated from embryos at embryonic days 12.5–14.5 and were maintained in DMEM supplemented with 15% FBS, 100 µg/ml penicillin G, and 100 µg/ml streptomycin. Primary astrocytes were prepared as described previously with minor modifications. In brief, cerebral cortex was prepared with smooth fine forceps from 2-day-old mice and was trypsinized for 12 min at 37°C, seeded on PDL-coated flasks, cultured with DMEM containing 10% FBS (GIBCO). Astrocytes that were grown to confluence 10–14 days after plating were divided and used for experiments (25). Primary lung epithelial cells were isolated from WT or Stk24^h/h^ mice as described by Wang et al. Cells were maintained in BMGM (Lonza) supplemented with 10% FBS, 100 µg/ml penicillin G, and 100 µg/ml streptomycin (26). HEK293T cells and HeLa cells were transfected with plasmids using PEI according to the manufacturer’s protocol. HeLa cells and A549 cells were transfected with Stk24 targeted siRNA (CAGGGUUUGUCAUUAAUAAdTdT and GCUCCGCACUAGAUCUAUUdTdT) using INTERFERin@ according to the manufacturer’s instructions.

### Quantitative Real-Time PCR and ELISA

Total RNA was extracted using TRIzol reagent (Invitrogen) following the manufacturer’s protocols, the cDNAs were synthesized using cDNA synthesis kit (Takara) following the manufacturer’s instructions, and real-time PCR was conducted using SYBER Green (Takara). The primer sequences were showed in Table S1 in Supplementary Material. The productions of pro-inflammatory cytokines and chemokines were detected by ELISA Kit (eBioscience) following the manufacturer’s instructions.

### Co-Immunoprecipitation and Immunoblot

Co-immunoprecipitation and immunoblot analysis were performed as previously reported (27). Transfected HEK293T cells were lysed in lysis buffer (20 mM Tris, pH 7.4, 150 mM NaCl, 1 mM EDTA, 10% glycerol, 1% NP-40, and a cocktail of proteinase inhibitors). Supernatants from cell lysates were incubated with HA-conjugated beads (Abmart) or M2 beads (Sigma) for 3–4 h. Endogenous protein was immunoprecipitated using specific antibodies plus protein A/G agarose. The beads were boiled at 100°C for 10 min, and the samples were analyzed by western blot.

### GST-Tag or His-Tag Pull-Down Assay

The fusion proteins of GST-null, GST-TAK1, GST-Stk24, MBP-Stk24-His, MBP-EGFP-His, sumo-IKKα-His, MBP-IKKβ-His, and sumo-IKKγ-His were expressed in *E. coli* BL21 strain and purified according to standard protocols with pre-equilibrated glutathione–Sepharose beads or with nickel-NTA beads (GE Healthcare). For GST-tagged pull-down assay, approximately 1 µg GST-fusion proteins (GST-null or GST-TAK1) were mixed with 20 µl pre-cleared anti-GST-coupled beads in 500 µl reaction medium, followed by adding 1 µg target purified protein (MBP-Stk24) and incubating at 4°C for 3 h with gentle rotation. For His-tagged pull-down assay, approximately 1 µg His-fusion proteins (MBP-EGFP-His, sumo-IKKα-His, MBP-IKKβ-His, or sumo-IKKγ-His) were mixed with 20 µl pre-cleared anti-His-coupled beads in 500 µl reaction medium, followed by adding 1 µg target purified protein (GST-Stk24) and incubating at 4°C for 3 h with gentle rotation. Precipitates were extensively washed three times with cell lysis buffer and then eluted with two loading buffer, boiled and then subjected to western blot.

### *In Vitro* Kinase Assay

*In vitro* kinase assay was performed as described previously (16, 28, 29). Briefly, GST-IκBα (1–54) plasmid was a kind gift from Xia’s Lab (Life Sciences Institute of ZJU, LSI). And GST-IκBα (1–54) recombinant protein was purified from *E. coli* BL21 strain. IKK complex was immunoprecipitated with antibody against IKKγ in HeLa cells treated with scrambled siRNA, or Stk24-specific siRNA, or in HeLa cells overexpressed with Flag-Stk24, Flag-Stk24-KR, or Flag-EGFP. 1 µg immunoprecipitated protein and 1 µg IκBα protein as a substrate were incubation at 30°C for 1 h under ATP buffer containing 50 mM Tris–HCl, pH 7.5, 2 mM ATP, 5 mM MgCl_2_, and the subjected to immunoblotting analysis for indicated antibodies.

### IL-17- or IL-1β-Induced Peritoneal or Pulmonary Inflammatory Responses

Age- and sex-matched WT or Stk24^h/h^ littermates were injected intraperitoneally with IL-17 (0.5 µg per mouse), or IL-1β (1.5 µg per mouse), or PBS. 24 h later, peritoneal mesothelial cells were isolated as described before (15). To remove intraperitoneal leukocytes, the peritoneal cavity was washed with 5 ml PBS. Then the peritoneal cavity was injected with 5 ml 0.25% trypsin. After 10 min, the trypsin solution was collected, and peritoneal cavity was washed with 5 ml DMEM containing 10% FBS. Cells were counted and then were stained with anti-Gr-1 and anti-CD11b antibodies, followed by flow cytometry. The expression of pro-inflammatory cytokines and chemokines in the cells was measured by real-time PCR analysis.

For IL-17-induced pulmonary inflammation, age- and sex-matched WT or Stk24^h/h^ littermates were treated with IL-17 (1 µg per mouse in 50 µl PBS) or PBS (50 µl) by intranasal injection. Then, 24 h later, PBS (1 ml) was used to obtain bronchoalveolar lavage fluid (BALF) though the trachea. Supernatants were used for ELISA, and precipitates were analyzed as lung-infiltrating cells. The residual lung-infiltrating cells were collected with 1 ml PBS wash. Cells were counted and then were stained with anti-Gr-1 and anti-CD11b antibodies, followed by flow cytometry. Lung tissues were collected in 1 ml ice-cold TriZol for subsequent real-time PCR analysis or in 4% paraformaldehyde for subsequent embedment in paraffin and staining with hematoxylin and eosin (H&E).

### Bone Marrow Chimeras

Recipient Stk24^h/h^ mice and control littermates were irradiated by sub-lethal dose of γ-ray (8.5cGy) to kill the bone marrow cells, 6 h post-irradiation, the recipients were injected with 1 × 10^7^ bone marrow cells from donor WT mice in the tail vein, respectively, which are WT → WT, Stk24^h/h^ → WT, and WT → Stk24^h/h^ groups. 8 weeks after bone marrow transplantation, mice’s blood were collected and determined with Stk24 genotyping analysis by PCR to exclude failure mice.

### Induction of EAE

Experimental autoimmune encephalomyelitis was induced by subcutaneous immunization with 200 µg MOG peptide (30–32) and 5 mg/ml heat-killed H37Ra strain of *Mycobacterium tuberculosis* emulsified in incomplete Freund’s adjuvant in the back and head region, followed by injection of pertussis toxin (PTX) at a dose of 200 ng/mouse in the tail vein. 48 h later, PTX was administered again for the second immunization. Disease severity was scored in a double-blinded manner by a trained observer without knowing the genotype of the mice or treatment protocols using a standard scale (16).

### *In Vitro* CD4^+^ T Cell Differentiation

*In vitro* CD4^+^ T cell differentiation was performed as previously described with little modification (16). Purified naive CD4^+^ T cells (CD44^lo^CD62L^hi^) were activated with 2 µg/ml of plate-bound anti-CD3 and 3 µg/ml anti-CD28 under different Th17 polarization conditions with or without IL-1β (10 µg/ml anti-IL-4, 10 µg/ml anti-IFN-γ, 20 ng/ml IL-6, and 5 ng/ml TGF-β, 20 ng/ml IL-1β), After 5 days of activation, the differentiated T cells were re-stimulated for 4 h with PMA and ionomycin in the presence of the protein transport inhibitor monensin (BFA), followed by intracellular staining of IL-17 to quantify the frequency of Th17 cells under different Th17 polarization conditions.

### Flow Cytometric and Histological Analyses

Cell suspensions were prepared as previously described (16) and subjected to flow cytometric analysis using a flow cytometer (BD). For histological analysis, lung tissue was fixed, perfused with 4% neutral formalin overnight, and paraffin-embedded sections were stained with H&E. Dissected spinal cords from mice were transcardially perfused with 4% neutral formalin and post-fixed overnight, and paraffin-embedded sections of spinal cords were stained with H&E to visualize leukocyte infiltration or with Luxol fast blue (LFB) to assess demyelination.

### Statistical Analysis

All data are expressed as the mean ± SEM except for Figure 2C shown as the mean ± SD. Statistical significance between two experimental groups was assessed using Student’s *t*-test. Statistical significance between more than two experimental groups was assessed using two-way ANOVA for multiple comparisons. A value of *P* < 0.05 was considered statistically significant.

## Results

### Stk24 Knockdown or Deficiency Inhibits IL-17-Induced Gene Expression of Pro-Inflammatory Cytokines and Chemokines

**Figure 1:**
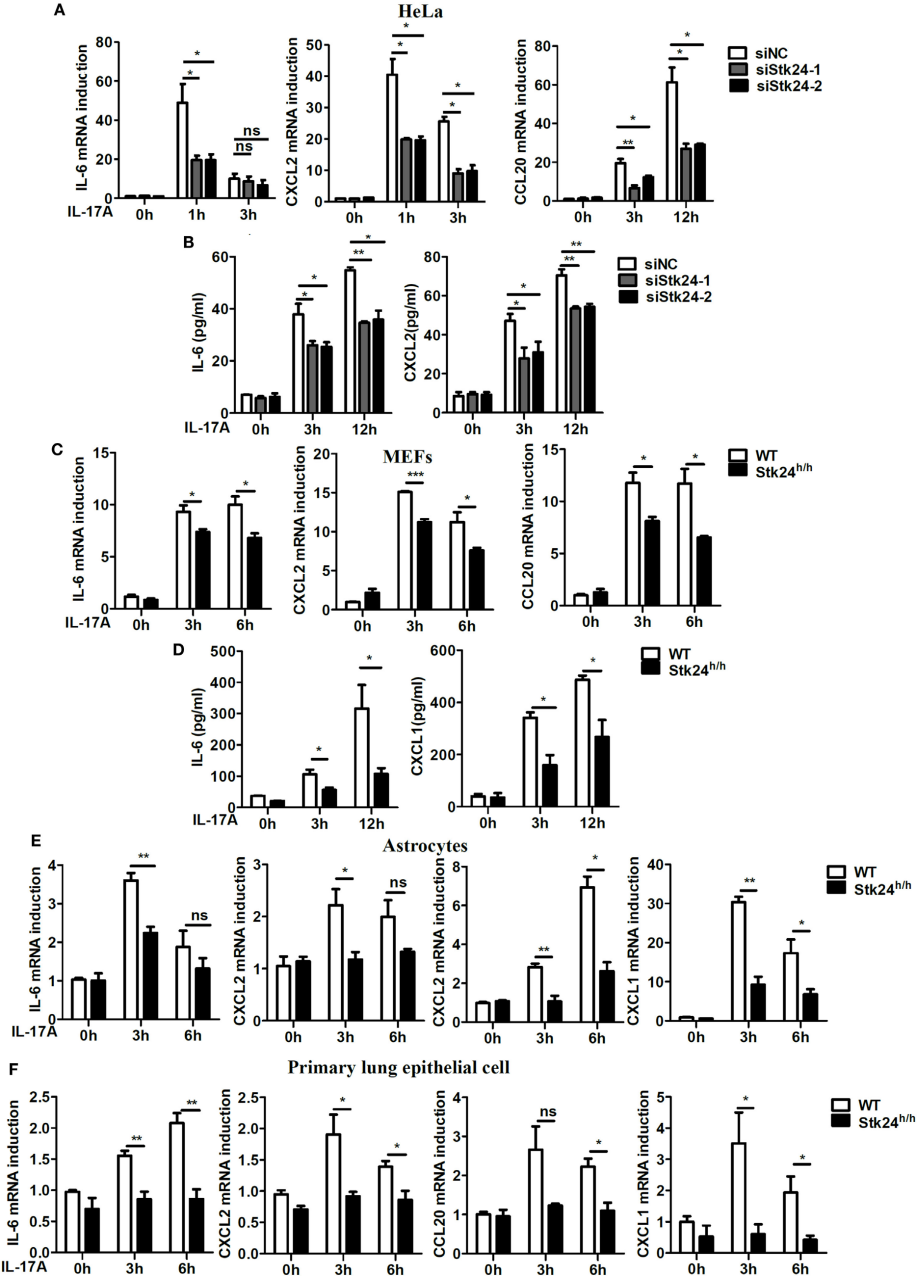
Serine/threonine kinase 24 (Stk24) deficiency suppresses interleukin 17 (IL-17)-induced pro-inflammatory cytokines and chemokines expression. **(A,B)** HeLa cells were transfected with Stk24 small interfering RNA (siRNA) or control siRNA and stimulated with IL-17 (50 ng/ml) for the indicated times. The mRNA levels and production of *IL-6, CXCL2*, and *CCL20* were analyzed by real-time PCR **(A)** and ELISA **(B)**, respectively. **(C,D)** Real-time PCR **(C)** and ELISA **(D)** analysis of *IL-6, CXCL2*, and *CCL20* mRNA expression and production in wild-type (WT) and Stk24-deficient (Stk24^h/h^) mouse embryo fibroblasts (MEFs) following stimulation with IL-17 (100 ng/ml) for the indicated times. **(E,F)** Real-time PCR analysis of *CXCL1, CXCL2, CCL20*, and *IL-6* mRNA expression in WT and Stk24^h/h^ primary astrocyte **(E)** and lung epithelial **(F)** cells following stimulation with IL-17 (100 ng/ml) for the indicated times (*P < 0.05, **P < 0.01, and ***P < 0.0001; Student’s *t*-test). Data are shown as the mean ± SEM of three independent experiments.

We first transfected a negative control siRNA or an Stk24-specific siRNA into HeLa cells to examine the role of Stk24 in IL-17-induced inflammatory cytokines and chemokines production. Compared with negative control siRNA-transfected HeLa cells, the IL-17-induced expression of IL-6, CXCL2, and CCL20 was significantly suppressed in Stk24-specific silenced HeLa cells (Figure 1A), and the protein production of IL-6 and CXCL2 also decreased (Figure 1B). Similarly, Stk24 silencing inhibited the IL-17-induced expression of IL-6, CXCL2, and CCL20 in human lung adenocarcinoma cells A549 (Figure S1A in Supplementary Material). Stk24-deficient (Stk24^h/h^) mouse is viable and shows no gross phenotypes (19). The Stk24 protein content in Stk24^h/h^ MEFs is greatly reduced (by more than 90%) (Figure S1B in Supplementary Material) as previous reported that the Stk24 protein content in Stk24^h/h^ neutrophils is reduced to less than 10%. We then examined the effect of Stk24 deficiency on the expression of IL-17-induced pro-inflammatory cytokines and chemokines in MEFs. Compared with WT MEFs, Stk24-deficient (Stk24^h/h^) MEFs exhibited much lower mRNA levels of IL-6, CXCL2, and CCL20 (Figure 1C) and protein levels of IL-6 and CXCL1 (Figure 1D). The expression of IL-17R was similar in Stk24-deficient (Stk24^h/h^) MEFs and WT MEFs (Figures S1B,C in Supplementary Material). Accordingly, Stk24 deficiency in primary astrocytes resulted in lower expression of IL-6, CXCL1, CXCL2, and CCL20 compared with WT astrocytes (Figure 1E). In addition, Stk24-deficient primary lung epithelial cells showed reduced induction of IL-17A-induced cytokines and chemokines (Figure 1F). Since IL-17F has similar functions as IL-17A, we further investigated the role of Stk24 in IL-17F-induced gene expression of pro-inflammatory cytokines and chemokines. As shown in Figures S1D,E in Supplementary Material, IL-17F-induced mRNAs expression of IL-6, CXCL2, and CCL20 was impaired in Stk24-silenced HeLa cells, as well as in Stk24-deficient MEFs. Furthermore, Stk24 knockdown in HeLa cells (Figures S1F,G in Supplementary Material) or Stk24 deficiency in primary MEFs (Figures S1H,I in Supplementary Material) resulted in the similar phonotype treated with TNF-α or IL-1β as that by IL-17 treatment, indicating the universal function of Stk24 in IL-17-, TNF-α-, or IL-1β-induced inflammation *in vitro*. Taken together, these results indicate that Stk24 positively regulates IL-17-induced inflammation *in vitro*.

### Stk24 Promotes IL-17-Induced Gene Expression of Pro-Inflammatory Cytokines and Chemokines in a Kinase Activity-Independent Manner

**Figure 2:**
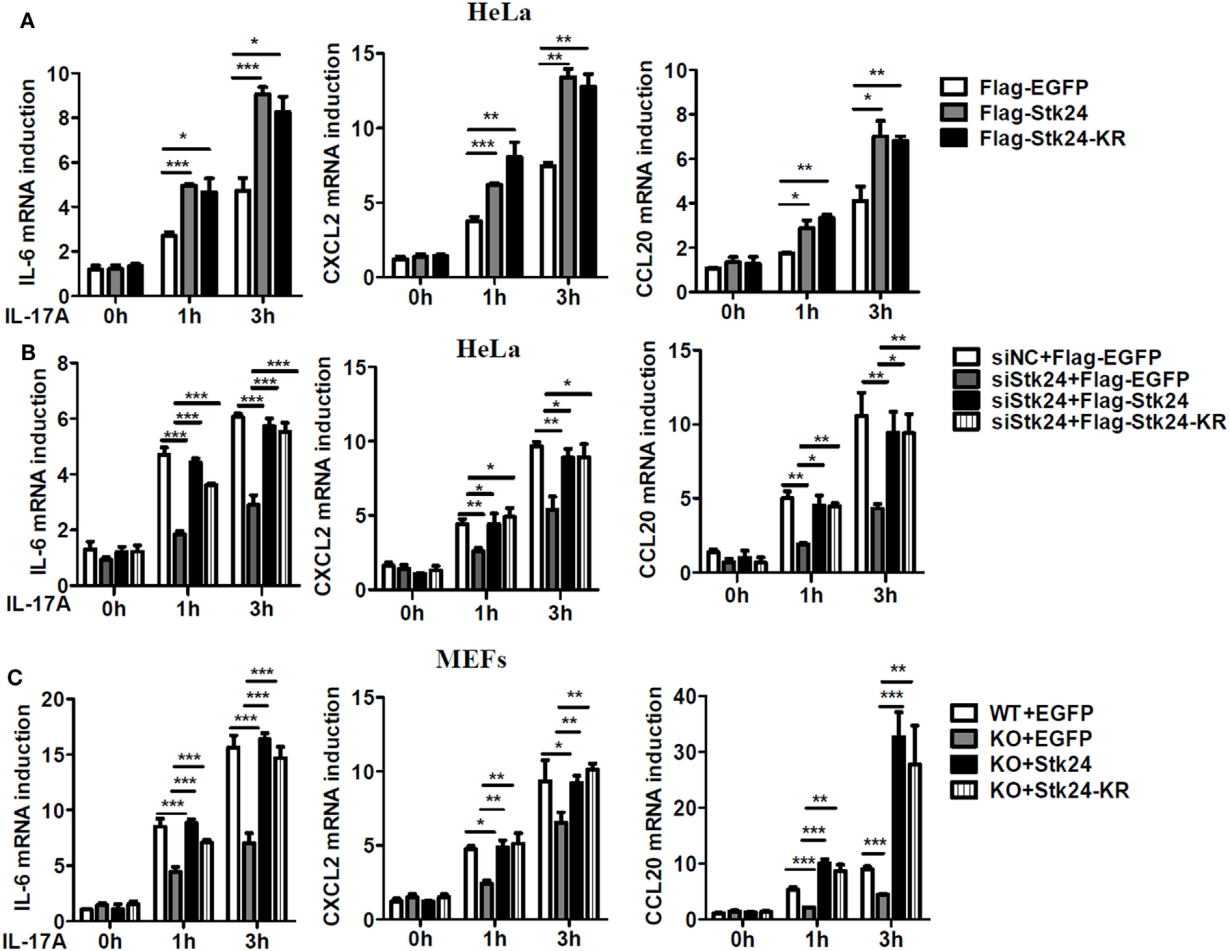
Serine/threonine kinase 24 (Stk24) promotes interleukin 17 (IL-17)-induced gene expression of pro-inflammatory cytokines and chemokines in a kinase activity-independent manner. **(A)** HeLa cells were transfected with Flag-EGFP, Flag-Stk24, or Flag-Stk24-K53R expressing plasmid and stimulated with IL-17 (50 ng/ml) for the indicated times. The mRNA levels of *IL-6, CXCL2*, and *CCL20* were analyzed by real-time PCR. **(B)** Real-time RT-PCR analysis of *IL-6, CXCL2*, and *CCL20* mRNAs in HeLa cells treated with scrambled siRNA or Stk24-specific small interfering RNA for 24 h and then overexpressed with Flag-EGFP, Flag-Stk24, or Flag-Stk24-KR. **(C)** Wild-type (WT) and Stk24^h/h^ mouse embryo fibroblasts (MEFs) infected with the control (EGFP), retrovirus expressing Flag-Stk24, or Flag-Stk24-K53R retrovirus were treated with IL-17 (100 ng/ml) for the indicated times. The mRNA levels and production of *IL-6, CXCL2*, and *CCL20* were analyzed by real-time PCR (*P < 0.05, **P < 0.01, and ***P < 0.0001; two-way ANOVA). Data are shown as the mean ± SEM of three independent experiments **(A,B)** and mean ± SD of two independent experiments **(C)**.

To further verify the role of Stk24 in IL-17-induced inflammation, a plasmid expressing Stk24 protein or its kinase dead mutant, Stk24-KR [a single-residue mutation at Lys53 (KR) in the catalytic site of Stk24] (33), was transfected into HeLa cells. As shown in Figure 2A, overexpression of Stk24 or its kinase dead mutant dramatically promoted IL-17-induced gene expression of IL-6, CXCL2, and CCL20, indicating that Stk24 kinase activation is not required for its function in IL-17-induced inflammation. Next, we overexpressed WT Stk24 or Stk24-KR in Stk24-silenced HeLa cells or Stk24-deficient MEFs to confirm the role of Stk24 in regulating IL-17-induced inflammation. As shown in Figure 2B, Stk24-silenced HeLa cells showed impaired gene expression of IL-6, CXCL2, and CCL20, which was rescued to a comparable level to the control group *via* overexpression of Flag-Stk24 or Flag-Stk24-KR. Stk24-deficient MEFs were transfected with a plasmid expressing Stk24 or Stk24-KR using a retroviral infection system. As shown in Figure 2C, restoration of Stk24 or Stk24-KR in Stk24^h/h^ MEFs rescued the IL-17-induced gene expression of IL-6, CXCL2, and CCL20 to a comparable level to WT MEFs. Therefore, our results suggest that Stk24 functions as a positive modulator of IL-17-induced inflammation in a kinase activity-independent manner.

### Stk24 Is a Critical Mediator of IL-17-Induced Inflammatory Responses *In Vivo*

**Figure 3:**
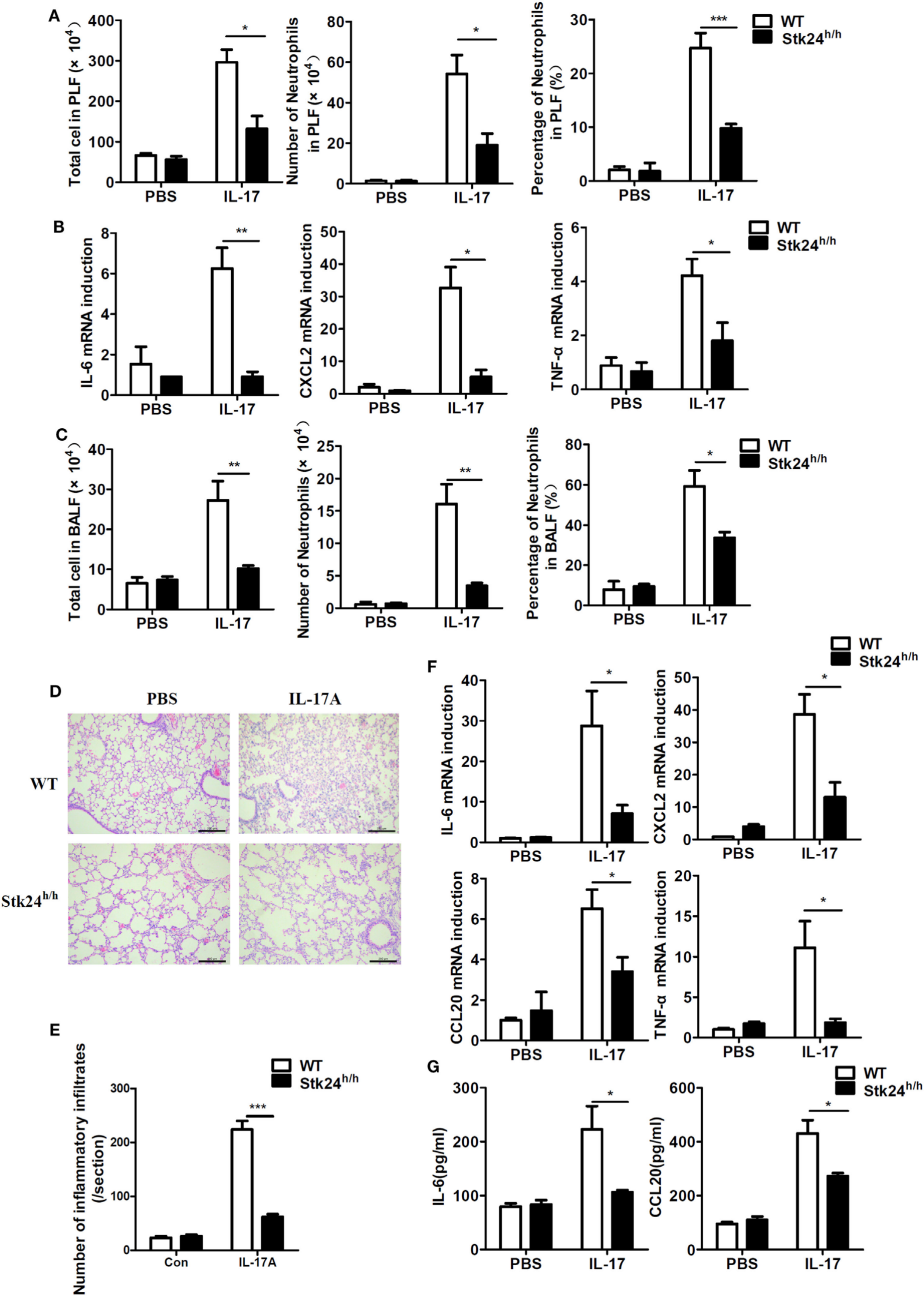
Serine/threonine kinase 24 (Stk24) deficiency inhibits interleukin 17 (IL-17)-induced inflammation *in vivo*. **(A,B)** Wild-type (WT) (*n* = 5) and Stk24^h/h^ (*n* = 5) mice were treated by intraperitoneal injection of PBS or IL-17 (0.5 µg in 200 µl PBS) for 24 h. The infiltration of total cells or neutrophils (Gr-1^+^CD11b^+^) into peritoneal lavage fluid was assessed by FACS **(A)**, and peritoneal mesothelial cells were isolated to determine *IL-6, CXCL2, CCL20*, and *TNF-α* mRNA expression **(B)**. **(C–G)** Infiltration of total cells or neutrophils (Gr-1^+^CD11b^+^) into the bronchoalveolar lavage fluid (BALF) of WT and Stk24^h/h^ mice (*n* = 5 per group) following intranasal injection of PBS (30 µl) or IL-17 (2 µg in 30 µl PBS), assessed 24 h after injection **(C)**. **(D)** Histology of lung tissue from mice treated as described in panel **(C)**. **(E)** Enumeration of the inflammatory infiltrates in lung tissue of panel **(D)**. **(F)** Lung tissue was examined for *IL-6, CXCL2, CCL20*, and *TNF-α* mRNA expression. **(G)** ELISA of IL-6, CCL20, and TNF-α in the BALF isolated from mice treated as described in panel **(C)** (*P < 0.05, **P < 0.01, and ***P < 0.0001; Student’s *t*-test). Data are shown as mean ± SEM of biological replicates. Similar results were obtained from two independent experiments.

The main function of IL-17 *in vivo* is to synergize local tissue inflammation by up-regulating pro-inflammatory and neutrophil-mobilizing cytokines and chemokines (15). To examine the *in vivo* effect of Stk24 in regulating IL-17-induced inflammation, Stk24-deficient (Stk24^h/h^) mice and control littermates (WT) were intraperitoneally injected with IL-17, and peritoneal lavage fluid (PLF) and expression of pro-inflammatory cytokines in peritoneal mesothelial cells were analyzed. There were significantly fewer total infiltrating cells and CD11b^+^Gr1^+^ neutrophils in PLF from Stk24^h/h^ mice than from WT mice (Figure 3A). Compared with peritoneal mesothelial cells isolated from WT mice, the gene expression of IL-6, CXCL2, and TNF-α mRNAs was significantly inhibited in Stk24^h/h^ mice (Figure 3B). However, Stk24 deficiency did not affect the IL-1β-induced the expression of IL-6 and CXCL2 (Figure S1J in Supplementary Material) and the infiltrating CD11b^+^Gr1^+^ neutrophils in PLF (Figure S1K in Supplementary Material) *in vivo*. These results indicate that Stk24 deficiency in mice impairs IL-17-induced peritoneal inflammation *in vivo*.

It is well-known that administration of IL-17 *via* the airway causes a severe pulmonary inflammation (14, 15). WT and Stk24^h/h^ mice were treated with intratracheal injection of PBS or IL-17 and sacrificed to analyze inflammatory cells in the BALF and lung inflammation. Upon IL-17 treatment, Stk24^h/h^ mice showed an obvious reduction in the number of total infiltrating cells and CD11b^+^Gr1^+^ neutrophils in the BALF compared with WT mice (Figure 3C). Histological analysis of lung tissue revealed less congestion of the alveolar lung tissues with scattered congestion of peribronchiolar and perivascular cells, as well as less inflammatory cells infiltration in Stk24^h/h^ mice compared with WT mice (*P* < 0.001) (Figures 3D,E). Consistent with the diminished inflammatory phenotype, Stk24^h/h^ mice showed reduced gene expression of IL-6, CXCL2, CCL20, and TNF-α in lung tissue (Figure 3F) and impaired protein production of IL-6 and CCL20 in the BALF compared with WT mice (Figure 3G). These results demonstrate that Stk24 deficiency in mice restricts IL-17-mediated pulmonary inflammation *in vivo*.

### Stk24 Deficiency in Non-Hematopoietic Cells Mediates the Reduced EAE Pathogenesis

**Figure 4:**
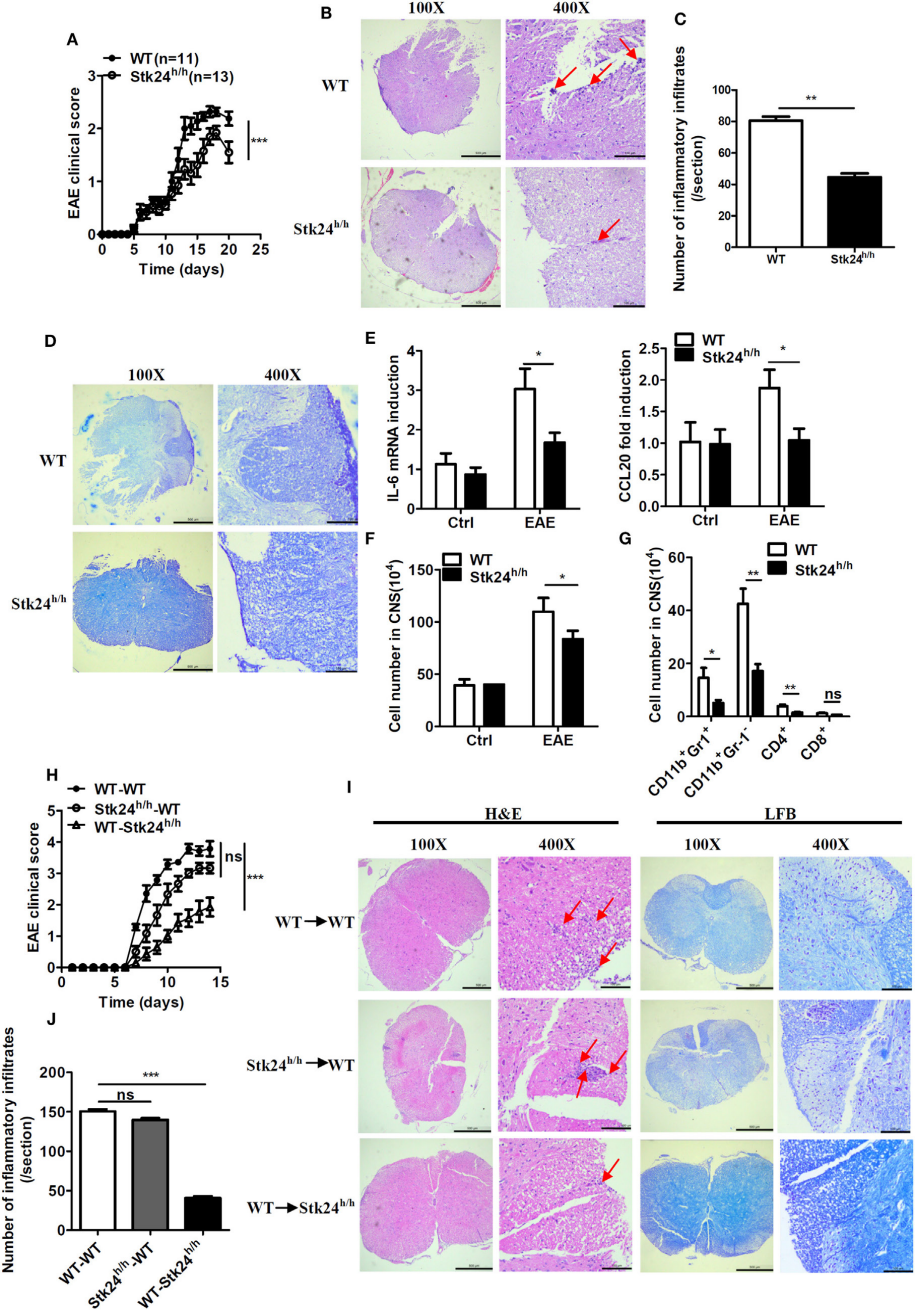
Serine/threonine kinase 24 (Stk24) deficiency in non-hematopoietic cells restricts the severity of experimental autoimmune encephalomyelitis (EAE). **(A–D)** Wild-type (WT) (*n* = 11) and Stk24^h/h^ (*n* = 13) mice were immunized with MOG (35–55) to induce EAE. Mean clinical scores were calculated every other day according to the standards described in Section “Materials and Methods.” **(A)**. Hematoxylin and eosin (H&E) and Luxol fast blue (LFB) staining of spinal cord sections from mice treated as described in panel **(A)** to visualize immune cell infiltration **(B)**. Enumeration of the inflammatory infiltrates of panel **(B) (C)** and demyelination **(D)**. **(E)** Relative mRNA expression levels of pro-inflammatory gene in the spinal cords of unimmunized and EAE WT and Stk24^h/h^ mice were determined by Q-PCR analysis. **(F,G)** Flow cytometric analysis of infiltrated cells of the immune response in the CNS of WT and Stk24^h/h^ EAE mice at day 14 after the second immunization as described in panel **(A)**, presented as the absolute number of cells. **(H,I)** Mean clinical scores after EAE induction in lethally irradiated WT-recipient mice adaptively transferred with WT or Stk24^h/h^ BM cells, and Stk24^h/h^-recipient mice adaptively transferred with WT BM cells **(H)** [WT → WT group (*n* = 7), Stk24^h/h^ → WT group (*n* = 6), and WT → Stk24^h/h^ group (*n* = 7)], followed by H&E and LFB staining of spinal cord sections to visualize immune cell infiltration and demyelination **(I)**. Enumeration of the inflammatory infiltrates of panel **(I) (J)**. Red arrows indicate alterations (inflammatory infiltrates and tissues damage) (*P < 0.05, **P < 0.01, and ***P < 0.0001) [Student’s *t*-test **(B–F)**, two-way ANOVA **(A,G)**]. Data are shown as mean ± SEM of biological replicates. Data are representative of two independent experiments.

Interleukin 17, which is associated with the pathogenesis of MS, plays a crucial role in the development of EAE (30). To explore the role of Stk24 in EAE pathogenesis, Stk24^h/h^ and WT mice were immunized with myelin oligodendrocyte glycoprotein [MOG; MOG (35–55)] and then intravenously injected with PTX to induce the EAE model. The severity of EAE pathology was notably alleviated in Stk24^h/h^ mice by reduced EAE clinical scores (Figure 4A), alleviated inflammation, including less tissue damage, inflammatory foci and perivascular cuff formation, and mononuclear cells’ infiltration (*P* < 0.01), as well as less demyelination in spinal cord (Figures 4B–D). Consistent with the lower EAE clinical scores, there was significantly lower gene expression of pro-inflammatory cytokines and chemokines in Stk24^h/h^ mice, including IL-6, CXCL2, and CCL20, in the brains and spinal cords compared with WT mice (Figure 4E). Flow cytometric analysis of mice CNS tissues (brains and spinal cords) demonstrated that the number of total cells and the number of CNS-infiltrating CD11b^+^Gr-1^−^ macrophages, CD11b^+^Gr-1^+^ neutrophils and CD4^+^ T cells were significantly reduced in Stk24^h/h^ mice compared with WT mice (Figures 4F,G). We next explored the cell compartment in which Stk24 contributes to EAE pathogenesis. Stk24^h/h^ and WT mice were lethally irradiated and reconstituted with bone marrow cells from WT or Stk24^h/h^ mice, respectively. After 8 weeks’ bone marrow transplantation, mice were subjected to MOG immunization and PTX injection to induce severe EAE. WT mice reconstituted with WT (WT →WT) or Stk24^h/h^ (Stk24^h/h^ → WT) bone marrow cells showed comparable EAE clinical scores, while Stk24^h/h^ mice reconstituted with WT bone marrow cells (WT → Stk24^h/h^) showed much lower EAE clinical scores compared with those from the other two groups (Figure 4H). H&E staining showed that tissue damage, inflammatory foci and perivascular cuff formation, and mononuclear cells infiltration were reduced in the spinal cords of the WT → Stk24^h/h^ group compared with the Stk24^h/h^ → WT or WT → WT group (*P* < 0.001) (Figures 4I,J), and impaired demyelination was also observed by LFB staining in the WT → Stk24^h/h^ group compared with the other two groups (Figure 4I). Collectively, these results demonstrate that Stk24 deficiency in non-hematopoietic cells impairs the development of EAE.

### Stk24 Promotes IL-17-Triggered NF-κB Activation

**Figure 5:**
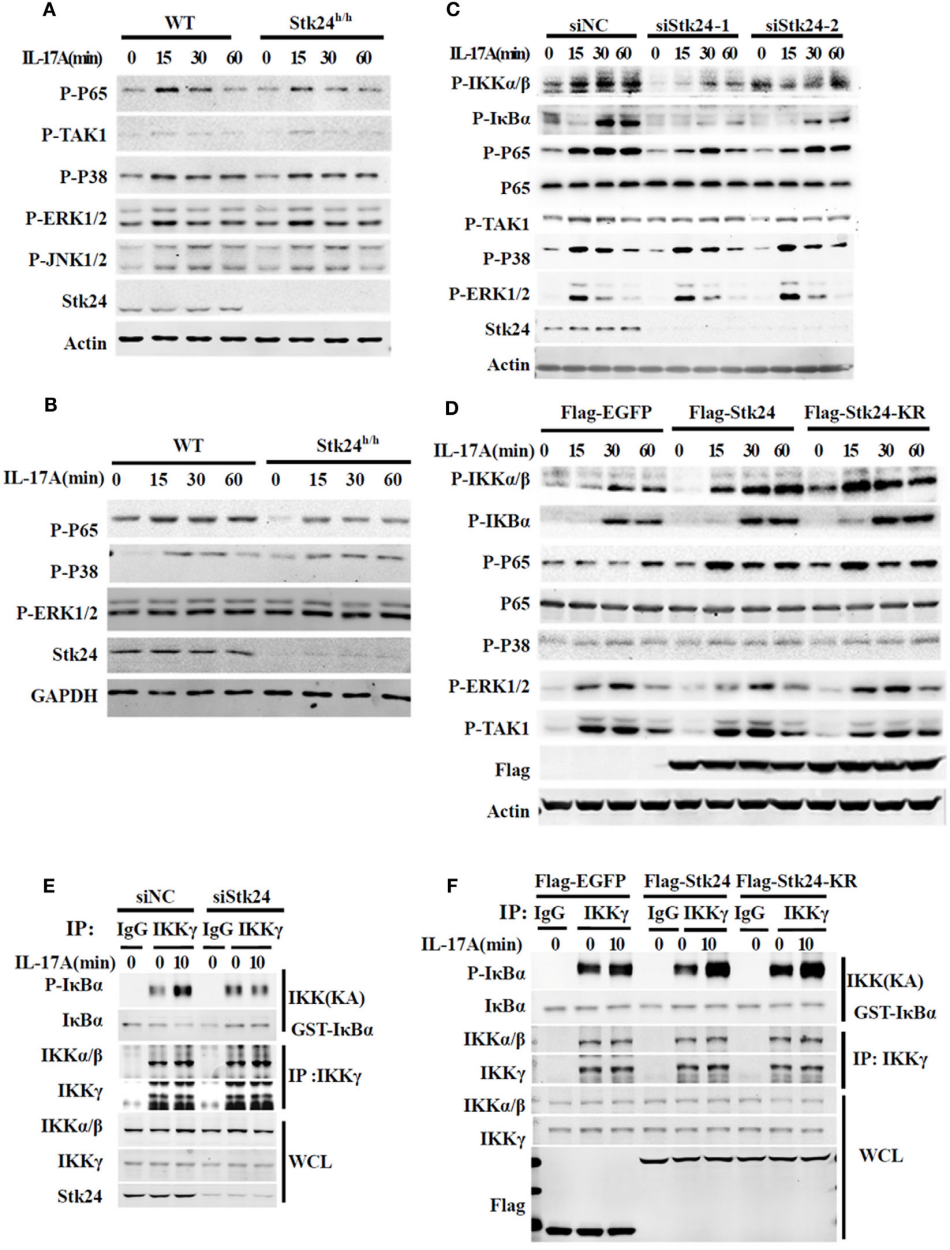
Serine/threonine kinase 24 (Stk24) promotes IL-17A-triggered activation of NF-κB independent of its kinase activity. **(A,B)** Wild-type (WT) and Stk24^h/h^ mouse embryo fibroblasts (MEFs) **(A)** or primary astrocytes **(B)** were treated with 100 ng/ml IL-17A for the indicated times. Whole-cell lysates were immunoblotted with the indicated antibodies. **(C)** HeLa cells were transfected with Stk24-specific small interfering RNA (siRNA) or control siRNA and stimulated with 50 ng/ml IL-17A for the indicated times. Immunoblot analysis probed with indicated antibodies. **(D)** HeLa cells were transfected the empty vector (Flag-EGFP) or plasmids encoding Flag-Stk24 or Flag-Stk24-K53R for 48 h and stimulated with 50 ng/ml IL-17A for the indicated times. Immunoblot analysis probed with indicated antibodies. **(E)** IKK complex was isolated from HeLa cells treated with scrambled siRNA or Stk24-specific siRNA by IP (using anti-IKKγ antibody) and subjected to IKK kinase assay using 1 µg GST-IκBα (1–54) as a substrate of IκB kinase assay. The GST-IκBα (1–54) recombinant protein was purified from bacteria described as in Section “Materials and Methods.” **(F)** IKK complex was isolated from HeLa cells overexpressed with Flag-EGFP, Flag-STK24, or Flag-STK24-K53R and by IP (using anti-IKKγ antibody) and subjected to IKK kinase assay using 1 µg GST-IκBα (1–54) as a substrate of IκB kinase assay. Similar results were obtained from three independent experiments.

We next explored the underlying molecular mechanism by which Stk24 regulates IL-17R signaling. IL-17-induced activation of the NF-κB and MAPK pathway is responsible for expression of pro-inflammatory cytokines and chemokines (8). We first examined IL-17-triggered NF-κB and MAPK activation in Stk24-deficient or knockdown cells. As shown in Figures 5A,B, Stk24 deficiency in astrocytes and MEFs inhibited IL-17A-induced phosphorylation of the NF-κB P65 subunit but had no effect on ERK1/2, P38 or TAK1 activation. Knockdown of Stk24 in HeLa cells (Figure 5C) and A549 cells (Figure S2A in Supplementary Material) remarkably inhibited phosphorylation of IKKα/β, IκBα, and P65 but not phosphorylation of TAK1 or MAPKs (including P38 and ERK) induced by IL-17 stimulation. Accordingly, the overexpression of Flag-Stk24 or Flag-Stk24-KR greatly increased IL-17A-induced phosphorylation of IKKα/β, IκBα, and P65 but not phosphorylation of TAK1 or MAPKs (Figure 5D). Meanwhile, both TNF-α- and IL-1β-activated signaling were reduced in Stk24 knockdown HeLa cells (Figures S2B,C in Supplementary Material) or Stk24-deficient MEFs (Figures S2D,E in Supplementary Material) compared with control cells. An *in vitro* kinase assay was performed to examine whether Stk24 affected IKK complex kinase activity. The IKK complex was isolated by immunoprecipitated with an anti-IKKγ antibody from Stk24-silenced HeLa cells treated with IL-17A for indicated times and the GST-IκBα (1–54) protein was purified from the *E. coli* BL21 strain as previous description (16, 29). The immunoprecipitated IKKγ and purified GST-IκBα (1–54) proteins were incubation at 30°C for 1 h with kinase assay buffer containing ATP and then subjected to immunoblotting analysis for indicated antibodies as previous description (16, 29). As shown in Figure 5E, the IKKγ kinase activity assay (KA) showed that silencing of Stk24 attenuated the IKK complex-induced IκBα phosphorylation *in vitro*. In contrast, the IKK complex isolated from Flag-Stk24- or Flag-Stk24-KR-overexpressed HeLa cells induced much stronger IκBα phosphorylation than that from Flag-EGFP-overexpressed HeLa cells (Figure 5F). Collectively, these results demonstrate that Stk24 promotes IL-17-induced IKK/NF-κB activation in a kinase activity-independent manner.

### Stk24 Directly Interacts With TAK1 and IKKβ

**Figure 6:**
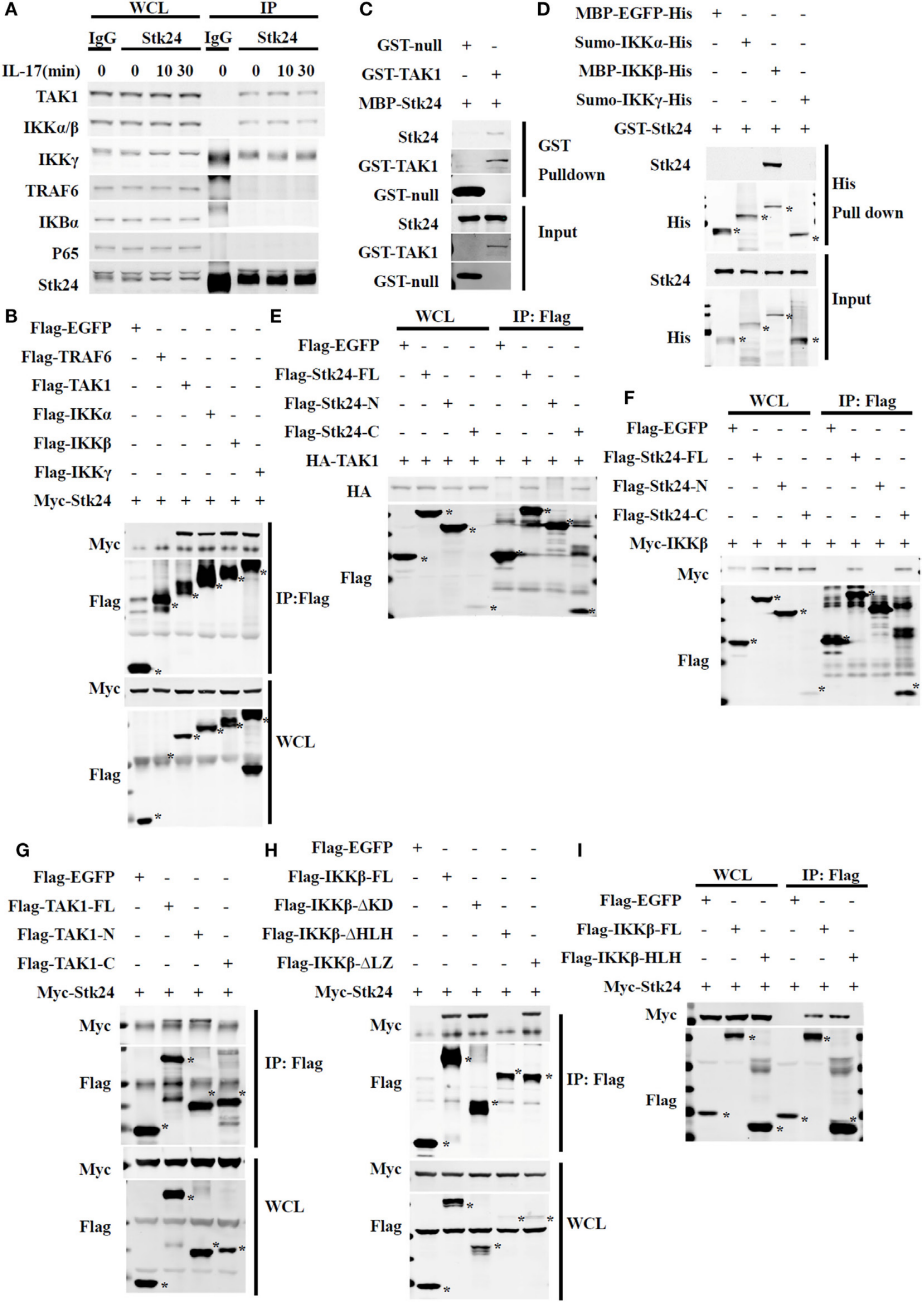
Serine/threonine kinase 24 (Stk24) directly associates with TAK1 and IKK in the IL-17R signaling pathway. **(A)** Immunoblot analysis of immunoprecipitates and whole-cell lysates of HeLa cells stimulated with human interleukin 17 (IL-17) (50 ng/ml) for the indicated times. Cells were subjected to immunoprecipitation with anti-IgG or anti-Stk24 antibody. **(B)** Immunoassay of HEK293T cells transfected with Myc-Stk24 and Flag-TRAF6 or Flag-TAK1, Flag-IKKα, Flag-IKKβ, Flag-IKKγ, or Flag-EGFP plasmids, followed by immunoprecipitation (IP) with an antibody to Flag (anti-M2) and immunoblot analysis with anti-Myc or anti-Flag. **(C)** MBP-Stk24, GST-TAK1, and GST-null proteins were expressed in BL21 cells and purified according to a standard protocol. GST-tagged fusion proteins were mixed with MBP-Stk24, respectively, and GST beads were added into pull-down buffer for the *in vitro* pull-down assay. Pull-down and input samples were separated by SDS-PAGE followed by immunoblotting with anti-GST or anti-Stk24 antibody. **(D)** MBP-EGFP-His, sumo-IKKα-His, MBP-IKKβ-His, sumo-IKKγ-His, and GST-Stk24 proteins were expressed in BL21 strain and purified according to the standard protocol. His-tagged fusion proteins were mixed with GST-Stk24, respectively, and nickel-NTA beads were added into pull-down buffer for the *in vitro* pull-down assay. Pull-down and input samples were separated by SDS-PAGE followed by immunoblotting with anti-His or anti-Stk24 antibody. **(E)** HEK293T cells were transfected to express HA-TAK1 together with Flag-EGFP, Flag-Stk24-FL, Flag-Stk24-N terminus, or Flag-Stk24-C terminus and subjected to IP with an antibody to Flag (anti-M2) and immunoblot analysis with anti-HA or anti-Flag. **(F)** HEK293T cells were transfected to express Myc-IKKβ together with Flag-EGFP, Flag-Stk24-FL, Flag-Stk24-N terminus, or Flag-Stk24-C terminus and subjected to IP with an antibody to Flag (anti-M2) and immunoblot analysis with anti-Myc or anti-Flag. **(G)** HEK293T cells were transfected to express Myc-Stk24 together with Flag-EGFP, Flag-TAK-FL, Flag-TAK1-N terminal, or Flag-TAK1-C terminal and subjected to IP with an antibody to Flag (anti-M2) and immunoblot analysis with anti-Myc or anti-Flag. **(H)** HEK293T cells were transfected to express Myc-Stk24 together with Flag-EGFP, Flag-IKKβ-FL, Flag-IKKβ-ΔKD, Flag-IKKβ-ΔLZ, or Flag-IKKβ-ΔHLH and subjected to IP with an antibody to Flag (anti-M2) and immunoblot analysis with anti-Myc or anti-Flag. **(I)** HEK293T cells were transfected to express Myc-Stk24 together with Flag-EGFP, Flag-IKKβ-FL, or Flag-IKKβ-HLH and subjected to IP with antibody to Flag (anti-M2). *Indicates the target band. Similar results were obtained from three independent experiments.

It is well-known that TAK1 mediates the activation of IKK/NF-κB in IL-17-triggered signaling (16, 31). We previously determined that Stk24 positively regulates IL-17-induced IKKα/β activation but not activation of its upstream kinase, TAK1. To further investigate the underlying mechanism of the positive regulation of the IKK pathway by Stk24, we immunoprecipitated endogenous Stk24 protein from the lysates of HeLa cells with or without IL-17 stimulation to determine which protein Stk24 interacts with. Stk24 physically associated with TAK1, IKKα/IKKβ, and IKKγ but not with TRAF6, IκBα, or P65 in HeLa cells (Figure 6A). HEK293T cells were transiently transfected with Flag-TAK1, Flag-IKKα, Flag-IKKβ, Flag-IKKγ, and Myc-Stk24 expression plasmids followed by immunoprecipitation with M2 (anti-flag) beads. As shown in Figure 6B, Stk24 interacted with TAK1, IKKα, IKKβ, and IKKγ but not with TRAF6. However, the GST pull-down assay showed that Stk24 directly interacted with TAK1 and IKKβ but not with IKKα or IKKγ (Figures 6C,D).

Serine/threonine kinase 24 contains an N-terminus [kinase domain (KD)] and C-terminus that retain Stk24 in the cytoplasm (33). To identify which regions of Stk24 are responsible for its interactions with TAK1 and IKKβ, we constructed two deletion mutants of Stk24: the N-terminal domain mutant (amino acids 1–313 of Stk24) and the C-terminal domain mutant (amino acids 314–431 of Stk24) (18) (Figure S3A in Supplementary Material). Co-immunoprecipitation experiments showed that only the C-terminal domain of Stk24 interacted with TAK1 (Figure 6E) and IKKβ (Figure 6F). TAK1 contains an N-terminus (KD) and a C-terminus, which is a high-probability coiled-coil domain (32, 34) (Figure S3B in Supplementary Material). Domain mapping experiments showed that Stk24 bound to the N-terminus of TAK1 (amino acids 1–303) (Figure 6G). IKKβ contains a KD, a leucine zipper (LZ), and a helix loop helix (HLH) (Figure S4C in Supplementary Material) (35). The different deletion constructs of IKKβ were mapped to Stk24 in HEK293T cells to determine the key domains that interact with Stk24. The co-immunoprecipitation assay showed that the IKKβ-ΔKD and IKKβ-ΔLZ deletion mutants but not the IKKβ-ΔHLH mutant immunoprecipitated with Stk24 (Figure 6H). We next constructed an HLH domain mutant (amino acids 577–756 of IKKβ) to confirm whether the IKKβ-HLH domain interacts with Stk24. As shown in Figure 6I, the IKKβ-HLH domain interacted with Stk24. Collectively, our results show that the Stk24 C-terminal domain directly interacts with the TAK1 N-terminal (KD) and IKKβ-HLH domains.

### Stk24 Promotes the IL-17-Induced Association of TAK1 With the IKK Complex

**Figure 7:**
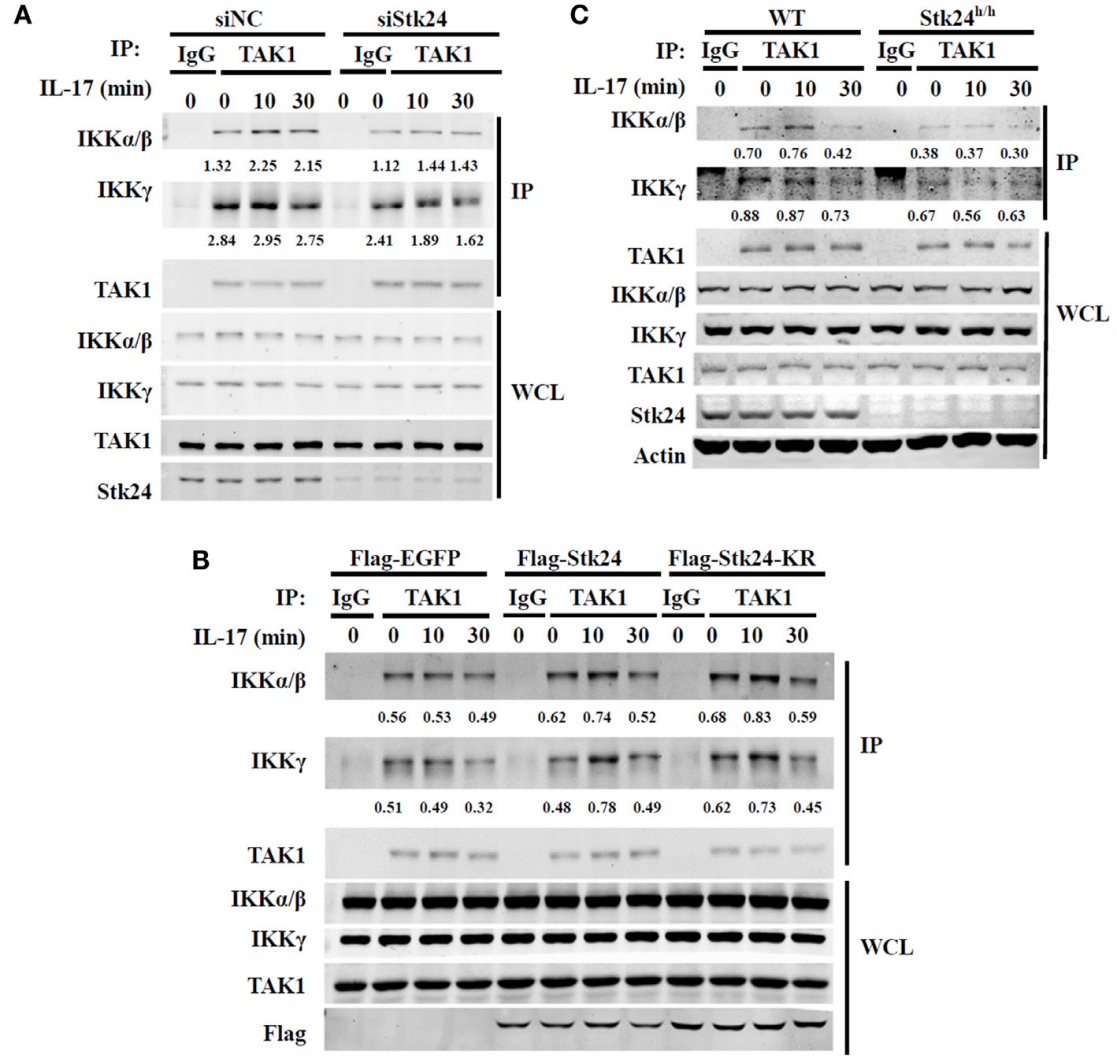
Serine/threonine kinase 24 (Stk24) promotes the interaction between TAK1 and IKKβ. **(A)** HeLa cells were transfected with control small interfering RNA (siRNA) or Stk24 siRNA. 48 h later, cells were stimulated with interleukin 17 (IL-17) (50 ng/ml) for the indicated times. Treated whole-cell lysates from HeLa cells were subjected to immunoprecipitation with anti-TAK1 or anti-IgG control antibody. The immunoprecipitated proteins were washed three times and subjected to immunoblot analysis with the indicated antibodies. **(B)** HeLa cells were overexpressed with Flag-EGFP, Flag-Stk24, or Flag-Stk24-KR for 36 h and stimulated for the indicated times with IL-17 (50 ng/ml). Treated whole-cell lysates from HeLa cells were then subjected to immunoprecipitation with anti-TAK1 or anti-IgG control antibody. **(C)** Wild-type (WT) and Stk24^h/h^ mouse embryo fibroblasts (MEFs) were stimulated for the indicated times with IL-17 (100 ng/ml). Treated whole-cell lysates from MEF cells were subjected to immunoprecipitation with anti-TAK1 or anti-IgG control antibody. The numbers under two blots (IKKγ or IKKα/β) are densitometric number of IKKγ or IKKα/β normalized to immunoprecipitated TAK1 protein. Similar results were obtained from three **(A,B)** or two **(C)** independent experiments.

It is well-known that TAK1 mediates the activation of IKK/NF-κB in IL-17-triggered signaling (16, 31). We previously reported that Stk24 affects IL-17-induced IKKα/β activation but not activation of its upstream kinase, TAK1, in a kinase activity-independent manner. Moreover, Stk24 directly binds with TAK1 and IKKβ. We next explored whether Stk24 functions as an adaptor to regulate the interaction of TAK1/IKK complexes. HeLa cells were transfected with an Stk24-specific siRNA or Stk24 expressing plasmids and co-immunoprecipitation assays were performed. The association between IKKα/β and TAK1 was increased by IL-17 stimulation, which was reduced by silencing Stk24 expression (Figure 7A; Figures S4A,B in Supplementary Material). By contrast, the association between IKKα/β and TAK1 was greatly enhanced in Stk24 or Stk24-KR overexpression HeLa cells versus control transfectants (Figure 7B; Figures S4C,D in Supplementary Material). To confirm these results, Stk24^h/h^ MEFs were subjected to immunoprecipitation with the anti-TAK1 antibody. The IL-17-induced association of TAK1 with the IKK complex was notably inhibited in Stk24^h/h^ MEFs compared with WT MEFs (Figure 7C; Figure S4E in Supplementary Material). Taken together, these results strongly suggest that Stk24 promotes the association of TAK1/IKK complex, which is required for IKK complex activation and downstream cytokines or chemokines induction.

## Discussion

The clarification of signaling mechanisms is critical for the development of new therapeutic strategies to treat IL-17-induced inflammation and IL-17-related autoimmune diseases. In this study, we identified a novel regulator of IL-17R signaling, protein kinase Stk24. We demonstrated that Stk24 promotes IL-17-induced inflammation *in vitro* and *in vivo*. Stk24-deficient mice are resistant to MOG (35–55)-induced EAE disease symptoms. We further showed that Stk24 directly binds with TAK1 and IKKβ and promotes the formation of the TAK1/IKKβ complex, leading to enhance IKKβ/NF-κB activation and downstream cytokines and chemokines expression.

Serine/threonine kinase 24, a member of the ste20 kinase family, regulates a wide range of fundamental cellular processes. Stk24 acts as an upstream regulator of NDR to control apoptosis, cell cycle, and growth (23). However, the role of Stk24 in specific signaling pathways, such as inflammation, remains unknown. Here, we determined that Stk24 positively regulates IL-17-induced inflammation in a kinase activity-independent manner. Stk24 deficiency in MEFs, lung epithelial cells, or astrocytes significantly reduced but not absolutely inhibited the expression of IL-17-induced pro-inflammatory cytokines and chemokines. Overexpression of Stk24 or its kinase dead mutant (Stk24-KR) not only significantly increased the IL-17-induced expression of pro-inflammatory cytokines and chemokines in HeLa cells but also restored the reduced induction of cytokine expression in Stk24-silenced HeLa cells and Stk24-deficent MEFs. Since Stk24^h/h^ mouse is not a complete knockout of Stk24 expression, there still were pro-inflammatory gene induction upon IL-17 stimulation in Stk24^h/h^ MEFs and astrocytes. Since it is possible that complete Stk24^h/h^ cells could still respond to IL-17A, Stk24 pathway might be partially involved in the IL-17R signaling. Therefore, Stk24 is a positive modulator of IL-17R signaling instead of being absolutely required for IL-17R signaling as other IL-17R signaling regulator (10, 14, 17, 36–38).

IL-17A or IL-17F binds to IL-17RA or IL-17RC, which is expressed on non-hematopoietic cells and hematopoietic cells, including epithelial cells, astrocytes, endothelial cells, fibroblasts, and some immune cells (1, 39). IL-17R signaling plays a crucial role in the development of EAE through non-hematopoietic cells, practically astrocytes (1, 10, 37). *In vivo* study demonstrated that Stk24 deficiency protects mice from EAE pathogenesis. Bone marrow reconstitution experiments further showed that Stk24 contributes to the development of EAE in a non-hematopoietic cells dependent manner. Stk24 deficiency did not effect on IL-1β mediated Th17 differentiation *in vitro* (Figure S5 in Supplementary Material). Since IL-1β or TNF-α-induced gene expression of cytokines and chemokines was also impaired in Stk24-deficient cells. It is unclear to what extent reduced EAE development in Stk24^h/h^ mice is due to impaired IL-17R signaling. Further experiments with abrogated IL-17R signaling activity should be performed to explore whether the effect of Stk24 deficiency on EAE development is due to the reduced IL-17R signaling rather than something else. Consistently with the *in vitro* data, Stk24^h/h^ mice expressed less IL-17-induced pro-inflammatory cytokines and chemokines than WT mice in IL-17 challenged inflammation, including peritonitis and pulmonary inflammation. Collectively, these data suggest that Stk24 positively regulates IL-17R triggered inflammation *in vivo* and *in vitro*, as well as IL-17 signaling-associated EAE pathogenesis in a non-hematopoietic cells dependent manner.

It has been well-documented that IL-17 binds to a unique receptor and triggers a signaling pathway that requires ubiquitination of TRAF6, leading to activation of NF-κB, MAPK cascades and expression of cytokines and chemokines (1–3). Previous studies have demonstrated Stk24 positively regulates the cell cycle, cell growth, migration and synapse development in a kinase activity-dependent manner in HEK293, COS-7, breast cancer cells (MDA-MB-231cells and A431 cells), or neurons (20–23). Stk24 promotes proliferation and tumorigenicity *via* the VAV2/Rac1 signaling axis as an adapter in MDA-MB-231cells (24). In hematopoietic cells, such as neutrophil, Stk24 functions as an important regulator of neutrophil degranulation by coordinating UNC13D-driven vesicle exocytosis (19). In our study, we first demonstrated Stk24 significantly promoted IL-17-induced activation of the IKKα/β/NF-κB signaling pathway but not the MAPKs pathway. Further co-immunoprecipitation experiments showed that Stk24 specifically interacted with TAK1/IKK complex and promoted the interaction of TAK1/IKK complex, leading to enhanced IKKα/β activation and downstream cytokines and chemokines production. The GST pull-down assay further confirmed that Stk24 directly binds with TAK1 and IKKβ but not with IKKα or IKKγ. Stk24^h/h^ MEFs or Stk24-silenced HeLa cells showed diminished NF-κB and decreased cytokines and chemokines production induced by IL-17F, which also signals *via* IL-17R–Act1–TRAF6–TAK1.

TNF-α and IL-1β are important cytokines which play crucial role in the inflammation, their receptors widely distribute on different cells, including hematopoietic cells and non-hematopoietic cells. TNF-α or IL-1β activates the TAK1/IKK complex *via* binding with its receptor, TNFR1/2 or IL-1R, respectively, and induces pro-inflammatory cytokines or chemokines expression to mediated inflammation (40–42). Here, we found Stk24 silencing and deficiency resulted in a significant impaired mRNA expression of cytokines and chemokines induced by TNF-α or IL-1β, as well as reduced NF-κB activation *in vitro*, indicating Stk24 is not a specific modulator of IL-17 responses, but a common modulator of inflammatory pathways induced by IL-17, TNF, and IL-1β. However, there was slightly reduced expression of cytokines and chemokines induced by IL-1β in Stk24^h/h^ mice compared with that in WT mice, which is inconsistent with the *in vitro* data that Stk24 deficiency in MEF and HeLa cells significantly inhibits the IL-1β-induced inflammation. Since the cell composition of peritoneal cavity is more complicated than that of *in vitro* system, Stk24 might function differently between hematopoietic cells and non-hematopoietic cells, the detail molecular mechanism needs to be further investigated.

In summary, our study identified a novel role for Stk24 in regulating IL-17-mediated signaling and inflammation, which is independent on its kinase activity. Stk24 promoted the association between TAK1 and the IKK complex, resulting in activation of IKKβ, which is required for NF-κB signaling and pro-inflammatory cytokines and chemokines expression. Stk24 deficiency in mice restricted IL-17-induced inflammation and EAE. These data show that Stk24 promotes IL-17-mediated inflammation and may represent a new potential therapeutic target for IL-17-related inflammation and autoimmune diseases.

## Ethics Statement

All animal experiments were performed in accordance with protocols approved by the Institutional Animal Care and Scientific Investigation Board of Zhejiang University (Authorized N.O. ZJU2015-040-01).

## Author Contributions

YJ, MT, WL, and XinyuanW performed the experiments. XiaojianW and YJ designed experiments and assisted in analyzing the data and edited the manuscripts.

## Conflict of Interest Statement

The authors declare that the research was conducted in the absence of any commercial or financial relationships that could be construed as a potential conflict of interest.
