# Supplementary material for: Protein Kinase Serine/Threonine Kinase 24 Positively Regulates Interleukin 17-Induced Inflammation by Promoting IKK Complex Activation
Source: Front Immunol. 2018 Apr 30;9:921. doi: 10.3389/fimmu.2018.00921 (PMC5936754; doi:10.3389/fimmu.2018.00921)

Protein Kinase Stk24 positively regulates IL-17-induced inflammation by promoting IKK complex activation

Yu Jiang, Miao Tian, Wenlong Lin, Xinyuan Wang, Xiaojian Wang*

Institute of Immunology, School of Medicine, Zhejiang University, Hangzhou, Zhejiang, 310058, China

*Correspondence: wangxiaojian@cad.zju.edu.cn;

Running title: Stk24 positively regulates IL-17 mediated inflammation

Key words: Stk24, EAE, IL-17, IKK, inflammation.

Address correspondence and reprint requests to: Dr. Xiaojian Wang, Institute of Immunology, Zhejiang University, 866 Yuhang Tang Road. Hangzhou 310058. Fax: (+86-571)-88208285. Phone: (+86-571)-88206268. e-mail: wangxiaojian@cad.zju.edu.cn.

Supplemental information

Fig. S1

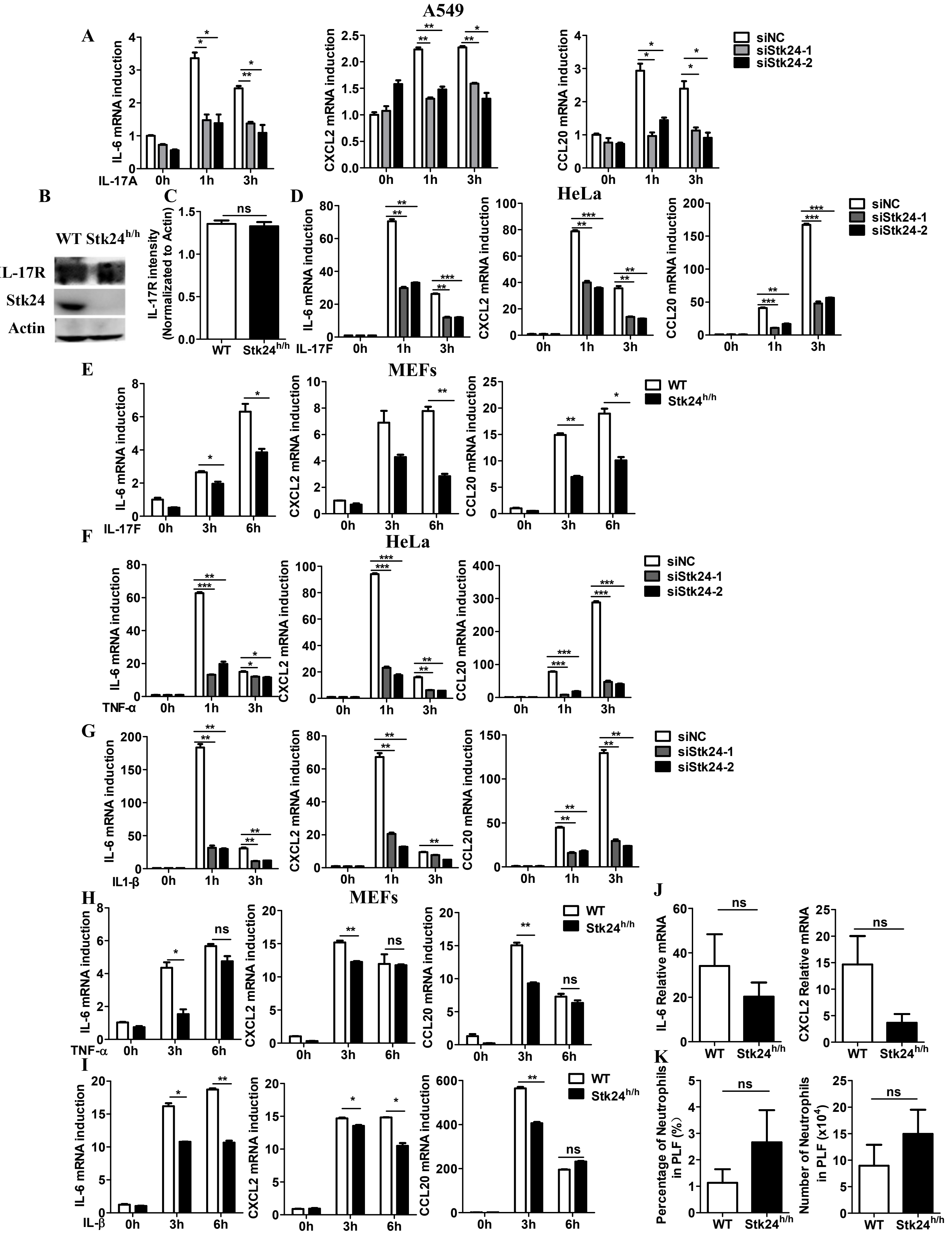

A549
HeLa
MEFs
HeLa
MEFs
siNC
siStk24-1
siStk24-2
WT
Stk24h/h
IL-17R
Stk24
Actin
ns

Fig. S1. Stk24 promotes IL-17A and IL-17F induced expression of pro-inflammatory cytokines.

(A) A549 cells were transfected with Stk24 siRNA or control siRNA, then stimulated with IL-17A (100 ng/ml) for indicated times. The mRNA level of *IL-6*, *CCL20*, and *CXCL2* were analyzed by real-time PCR**.** **(B, C)** WT and Stk24$^{h/h}$ MEFs were subjected to immunoblot analyzed with indicated antibodies **(B)**, densitometric analysis of IL-17R were performed by IL-17R protein normalized to Actin protein **(C)**. **(D)**HeLa cells were transfected with Stk24 siRNA or control siRNA, then stimulated with IL-17F (100 ng/ml) for indicated times. The mRNA level of *IL-6*, *CCL20*, and *CXCL2* were analyzed by real-time PCR**.** **(E)**wild-type (WT) and Stk24-deficient (Stk24$^{h/h}$) MEFs were treated with IL-17F (100 ng/ml) for indicated times, the induction of *CXCL2*, *CCL20* and *IL-6* mRNA expression were analyzed by real-time PCR. **(F, G)** HeLa cells were transfected with Stk24 specific siRNA or control siRNA, and then were treated with TNF-α (20 ng/ml) **(F)** or IL-1β (10 ng/ ml) **(G)** for indicated times, the induction of *CXCL2*, *CCL20* and *IL-6* mRNA expression were analyzed by real-time PCR. **(H, I)** WT and Stk24$^{h/h}$ MEFs were treated with TNF-α (20 ng/ml) **(H)** or IL-1β (10 ng/ ml) **(I)** for indicated times, the induction of *CXCL2*, *CCL20* and *IL-6* mRNA expression were analyzed by real-time PCR. **(J)** WT (n = 6) and Stk24$^{h/h}$ (n = 5) mice were treated by intraperitoneal injection of PBS or IL-1β (1.5 μg in 200 μl PBS) for 24 hours, and peritoneal mesothelial cells were isolated to detect *IL-6* and *CXCL2* mRNA expression. **(K)** The infiltration of neutrophils (Gr-1^{+}CD11b^{+}) into PLF was assessed by FACS, summary graph of the percentages of cells **(left)** and the absolute numbers of cells **(right)** in the PLF**.***P<0.05, **P<0.01 and ***P<0.0001, Ns means no significant difference (Student's t-test). Mean ± SEM of biological replicates. Similar results were obtained in two independent experiments.

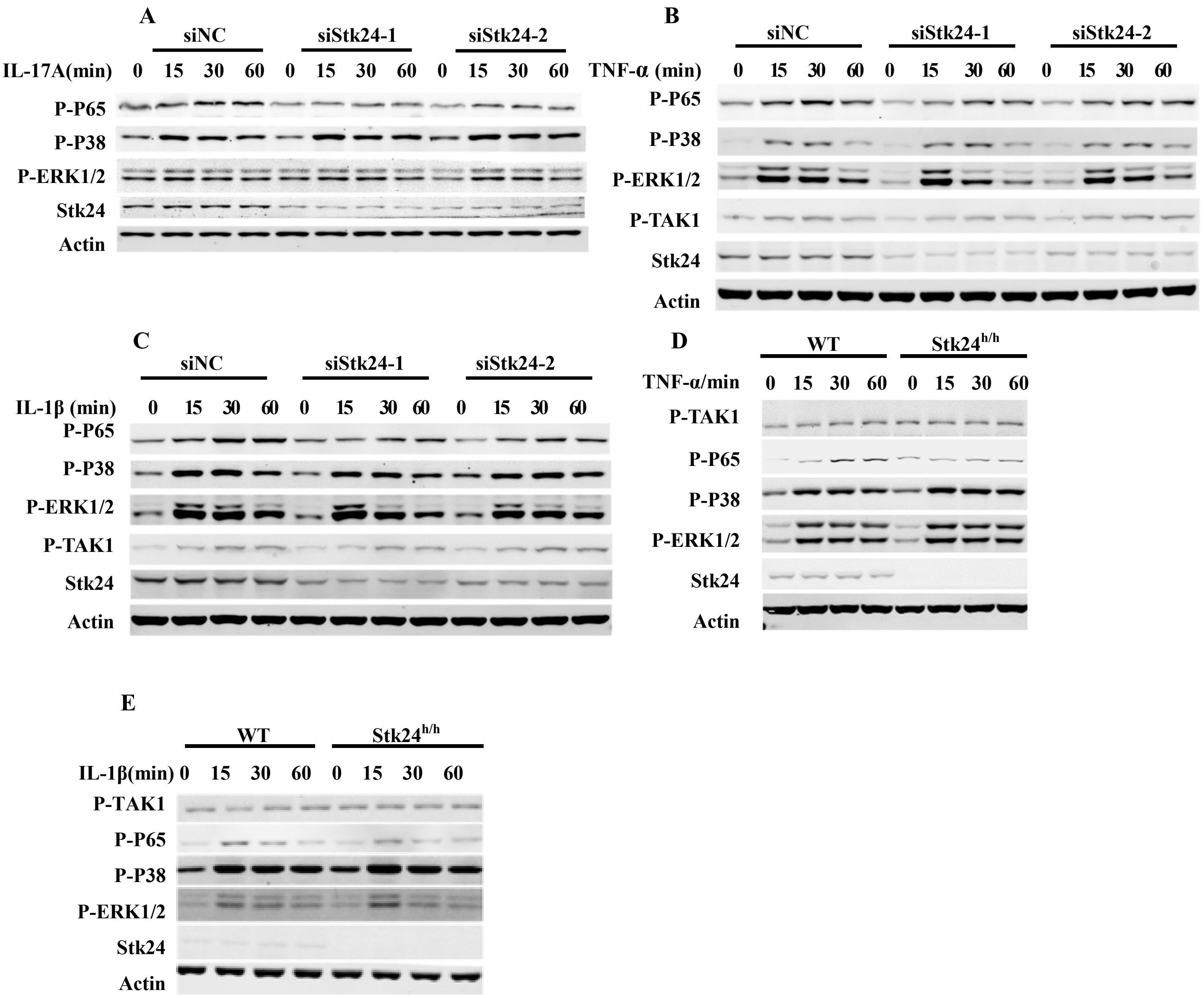

Fig. S2.Stk24 promotes IL-17A, IL-1β and TNF-α triggered activation of NF-κB.

(A) A549 cells were transfected with Stk24 specific siRNA or control siRNA, then were stimulated with 50 ng/ml IL-17A for indicated times. Immunoblot analysis probed with indicated antibodies. **(B, C)** HeLa cells were transfected with Stk24 specific siRNA or control siRNA, then were treated with TNF-α (20 ng/ml) **(B)** or IL-1β (10 ng/ ml) **(C)** for indicated times. Immunoblot analysis probed with indicated antibodies. **(D, E)** MEF cells were treated with TNF-α (20 ng/ml) **(E)** or IL-1β (10 ng/ ml) **(F)** for indicated times. Whole cell lysates were immunblotted with the indicated antibodies**.** Similar results were obtained in at least two independent experiments.

Fig.S3.

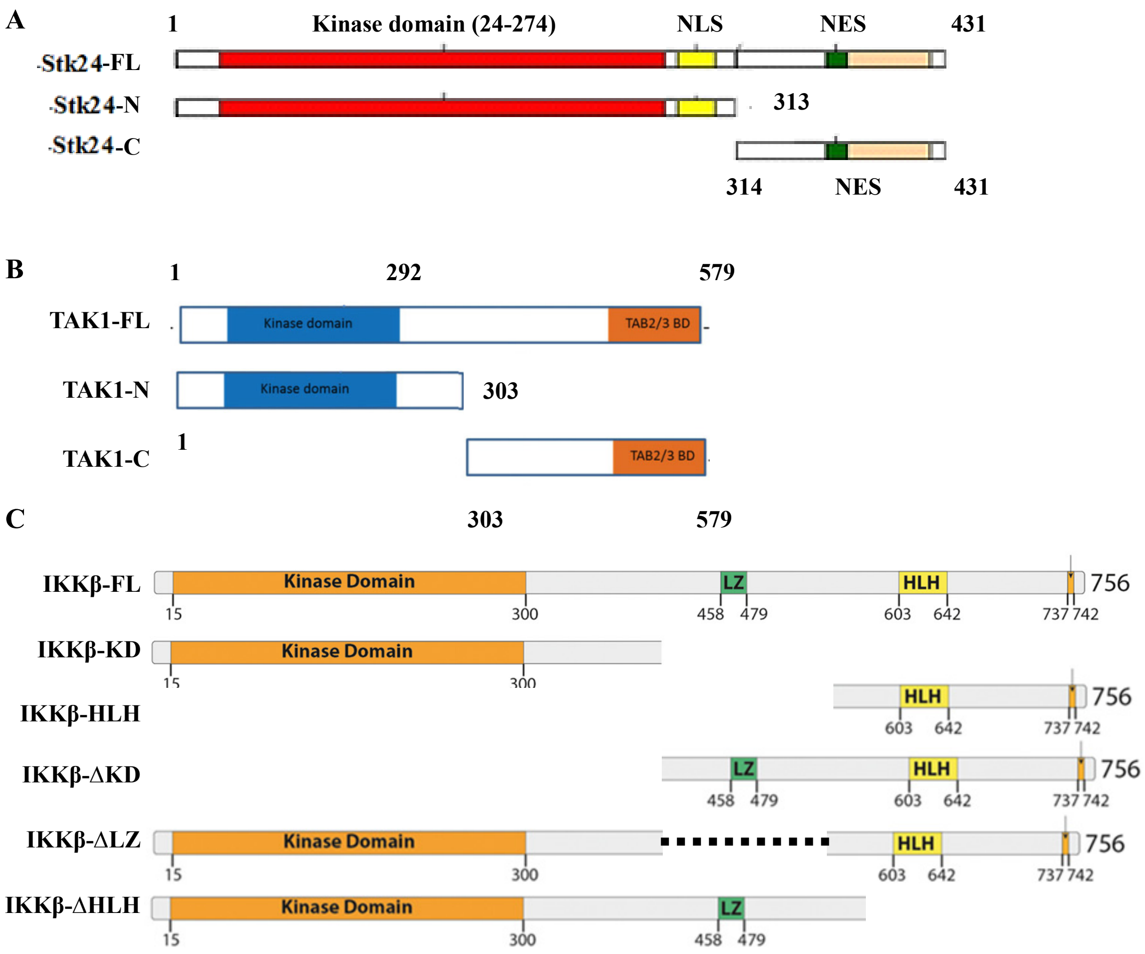

Fig. S3. Schematic diagram of Stk24, TAK1, and IKKβ deletion mutants. Schematic diagram of Stk24 **(A)**, TAK1 **(B)** and **(C)** deletion mutants.

Fig.S4.

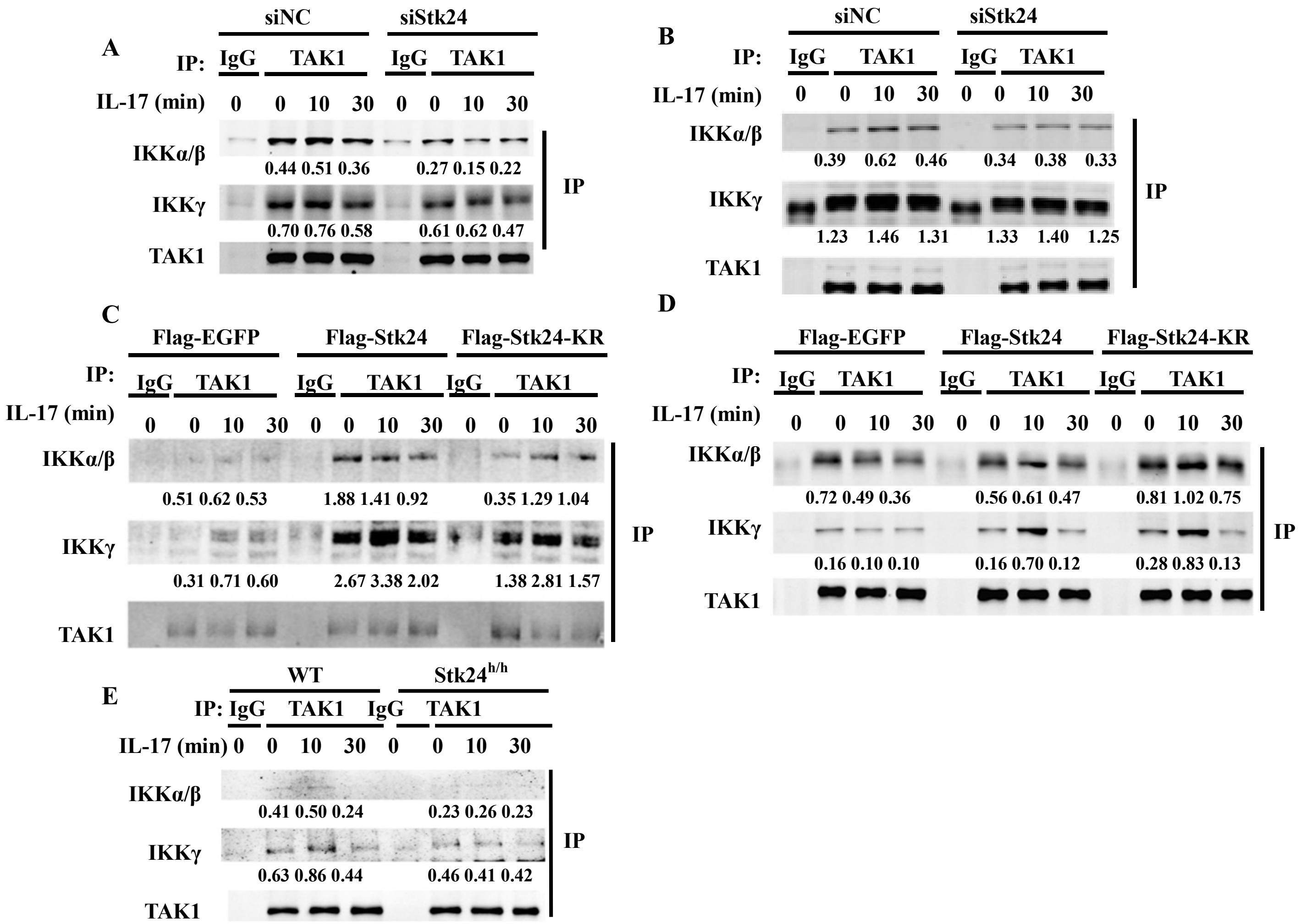

Fig. S4. Replicate data of "Stk24 promotes the interaction between TAK1 and IKKβ".

(A,B) HeLa cells were transfected with control siRNA or Stk24 siRNA. 48 hours later, cells were stimulated with IL-17 (50 ng/ml) for the indicated times. Treated whole-cell lysates from HeLa cells were subjected to immunoprecipitation with anti-TAK1 or anti-IgG control antibody. The immunoprecipitated proteins were washed three times and subjected to immunoblot analysis with the indicated antibodies. **(C, D)** HeLa cells were overexpressed with Flag-EGFP, Flag-Stk24, or Flag-Stk24-KR for 36 hours and stimulated for the indicated times with IL-17 (50 ng/ml). Treated whole-cell lysates from HeLa cells were then subjected to immunoprecipitation with anti-TAK1 or anti-IgG control antibody. **(E)**WT and $Stk24^{h/h}$ MEFs were stimulated for the indicated times with IL-17 (100 ng/ml). Treated whole-cell lysates from MEF cells were subjected to immunoprecipitation with anti-TAK1 or anti-IgG control antibody. The numbers under the two blots (IKKγ or IKKα/β) are densitometric number of IKKγ or IKKα/β normalized to immunoprecipitated TAK1 protein.

Fig.S5.

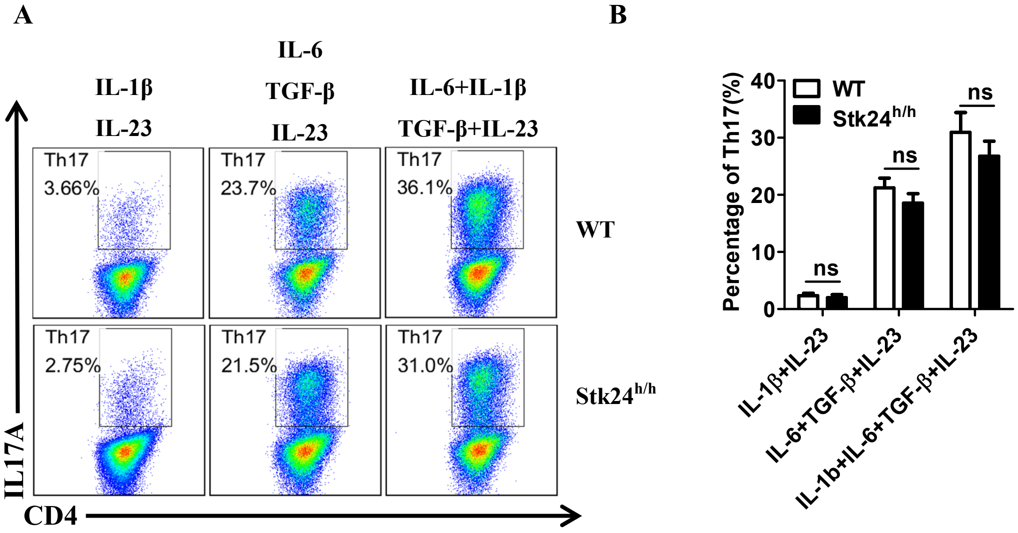

Fig. S5.IL-1β induced Th17 cells differentiation in WT and Stk24$^{h/h}$ CD4^{+} T cells. (A, B) The naïve CD4^{+} T cells were polarized under indicated Th17 differential conditions, respectively, 5 days later the CD4^{+} T cells were fixed and permeabilized, followed by flow cytometry to measure intracellular IL-17A. Data are presented as the representative plot **(A)** and summary graph of the percentages of cells **(B)**. Ns means no significant difference, (Student's t-test). Mean ± SEM of biological replicates. Similar results were obtained in two independent experiments.

Supplemental Table 1 Real-time PCR primers

humanβ-actin	Forward	GCA TCC TCACCC TGA AGTAC
	Reverse	TTCTCCTTAATGTCACCCAC
human IL-6	Forward	ATGAACTCCTTCTCCACAAGCGC
	Reverse	GGGAAGGCAGCAGGCAACAC
human CXCL2	Reverse	CTCAAGAATGGGCAGAAAGC
	Reverse	AAACACATTAGGCGCAATCC
human CCL20	Forward	GCGCAAATCCAAAACAGACT
	Reverse	CAAGTCCAGTGAGGCACAAA
mouse actin	Forward	CAAGTCCAGTGAGGCACAAA
	Reverse	CGTTGACATCCGTAAAGACC
mouse IL-6	Forward	AGTTGCCTTCTTGGGACTGA
	Reverse	TCCACGATTTCCCAGAGAAC
mouse CXCL2	Forward	CCTGGTTCAGAAAATCATCCA
	Reverse	CTTCCGTTGAGGGACAGC
human CCL20	Forward	AACTGGGTGAAAAGGGCTGT
	Reverse	GTCCAATTCCATCCCAAAAA

Supplementary material Full gel for figures

Fig5A

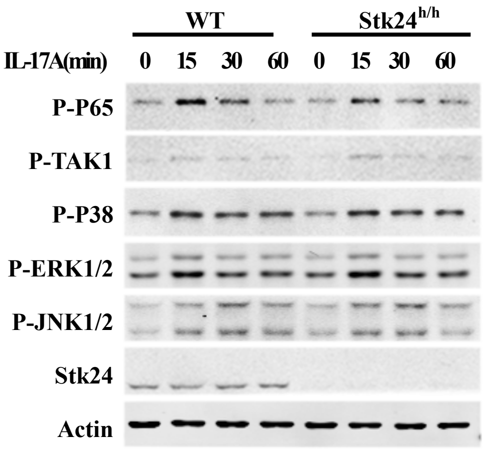

Full gel for Fig5A

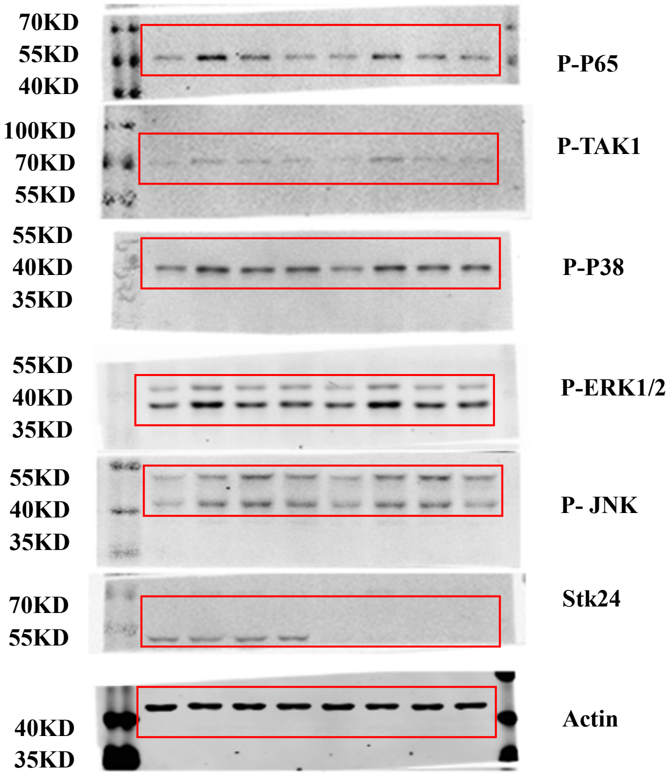

Fig5B

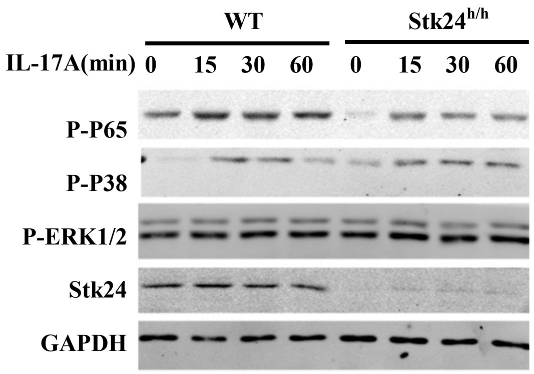

Full gel for Fig5B

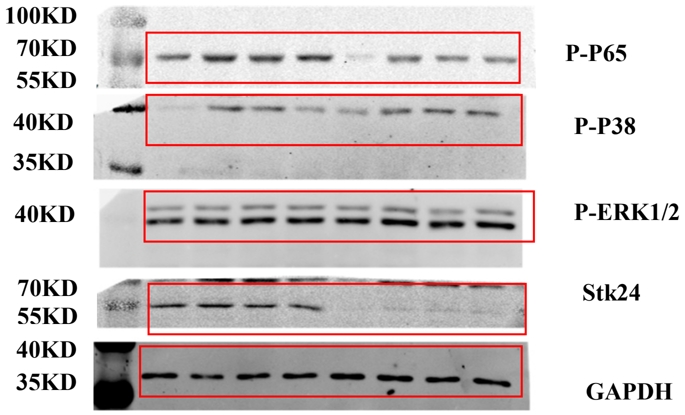

Fig5C

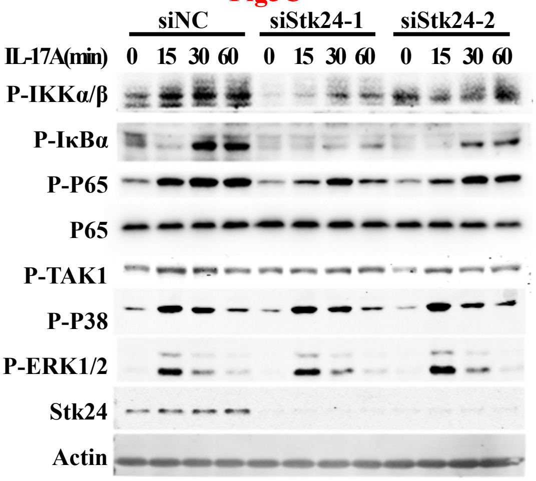

Full gel for Fig5C

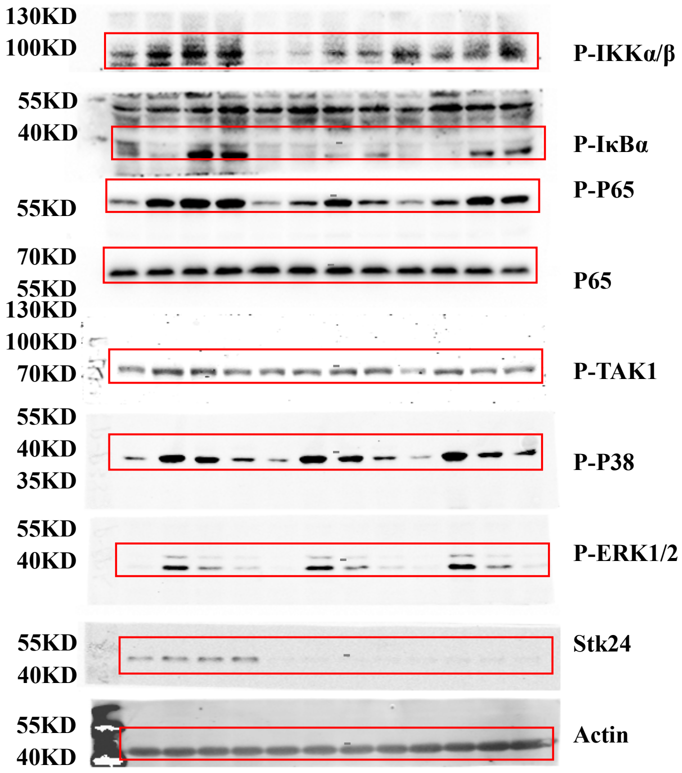

Fig5D

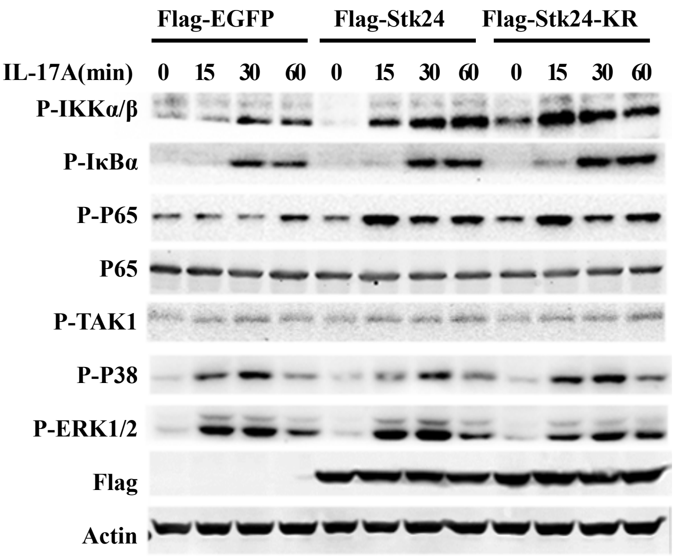

Full gel for Fig5D

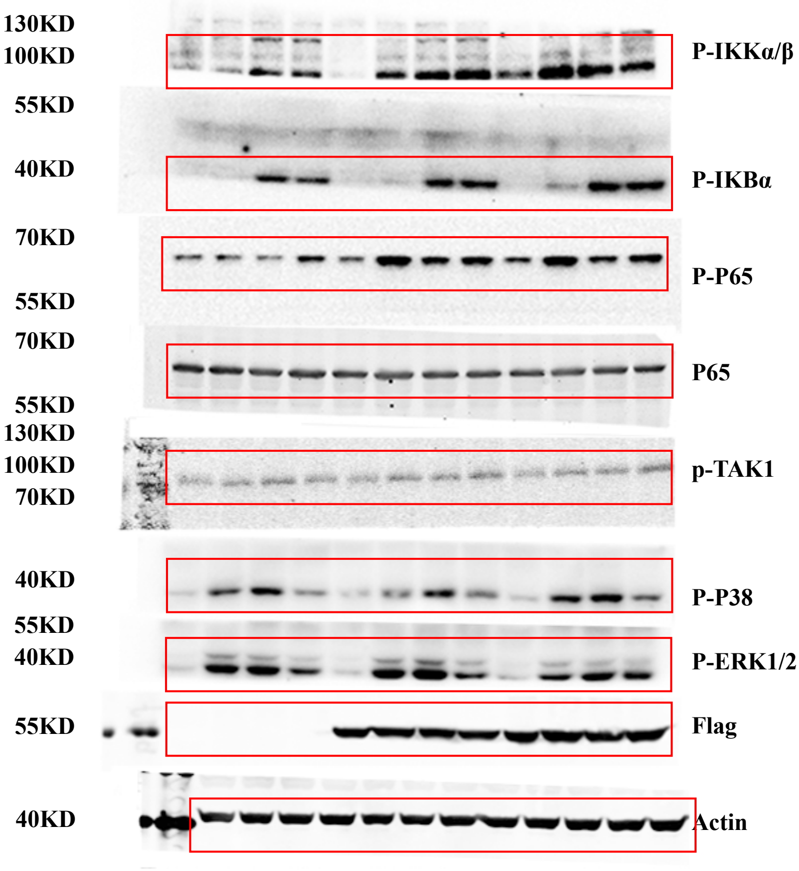

Fig5E

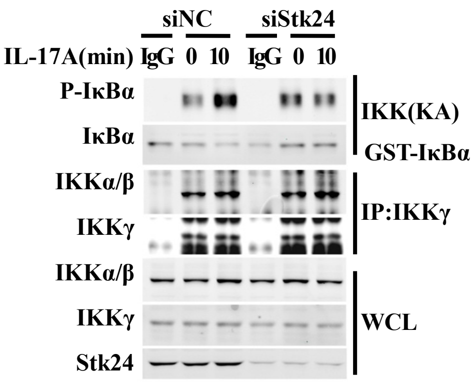

Full gel for Fig5E

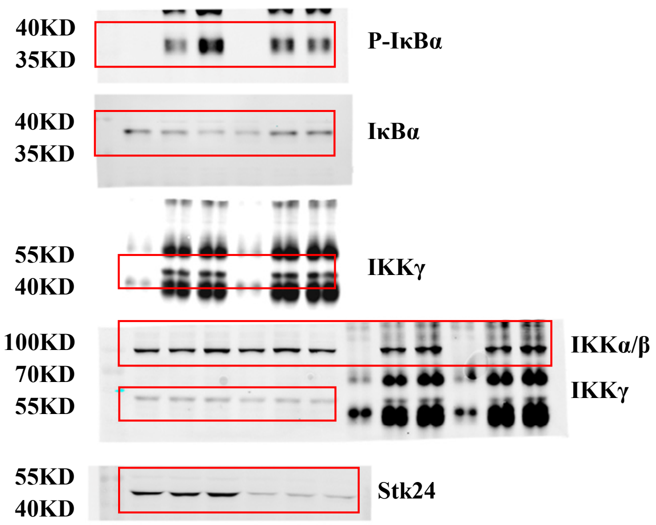

Fig5F

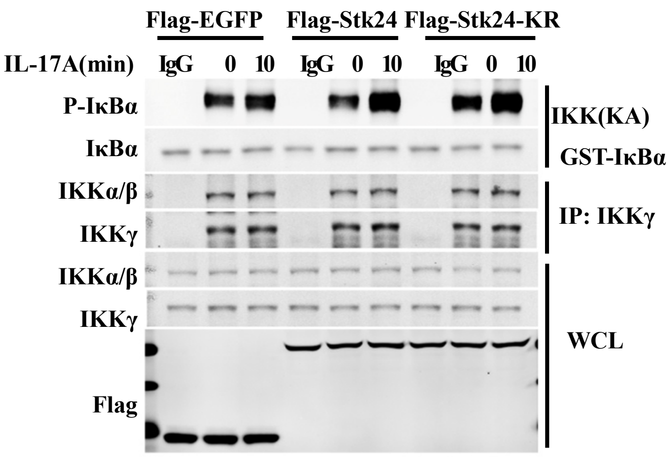

Full gel for Fig5F

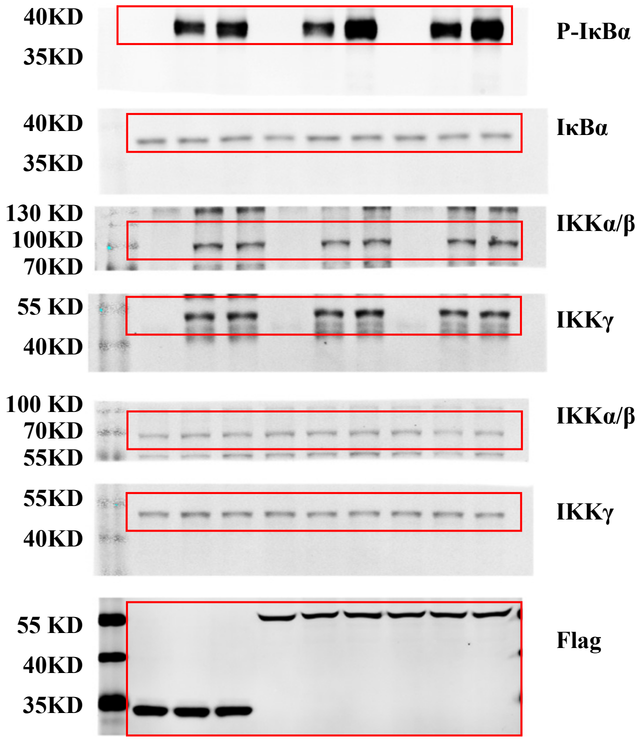

Fig6A

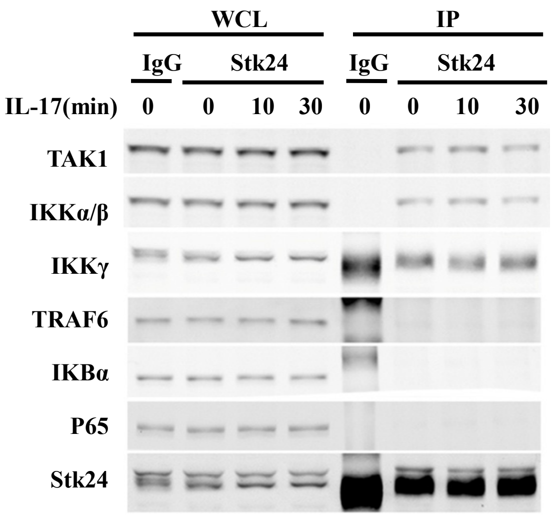

Full gel for Fig6A

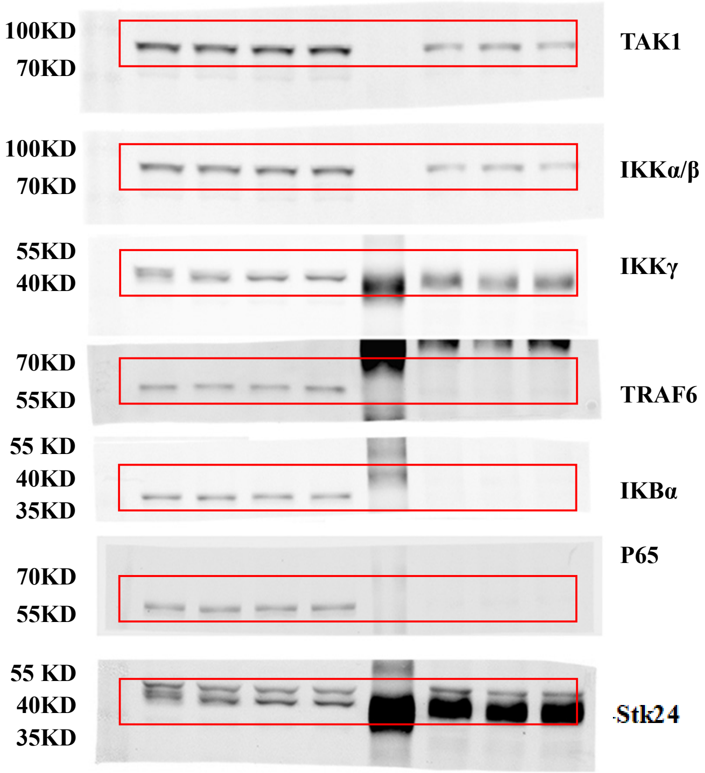

Fig6B

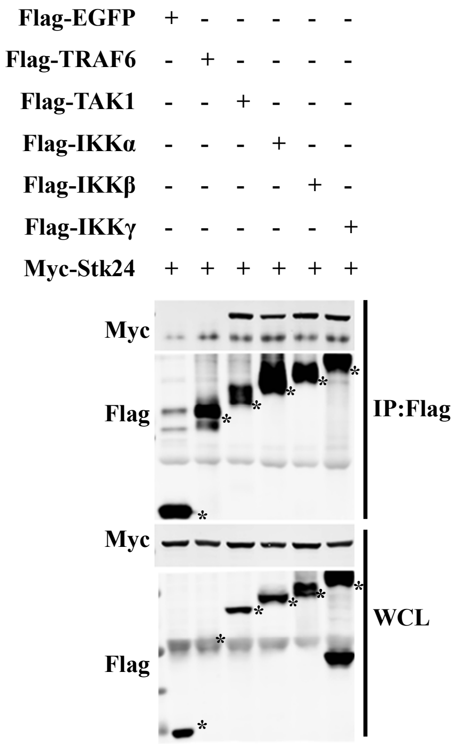

Full gel for Fig6B

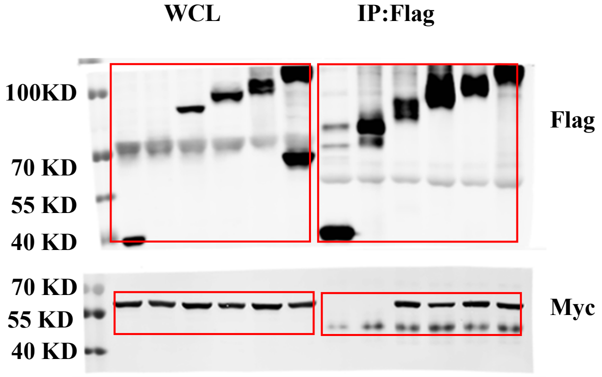

Fig6C

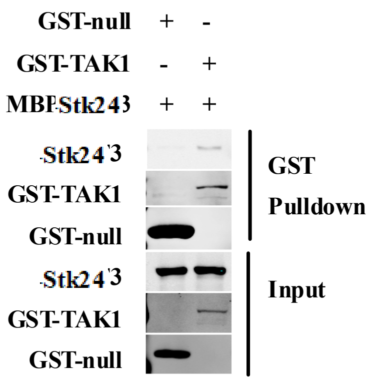

Full gel for Fig6C

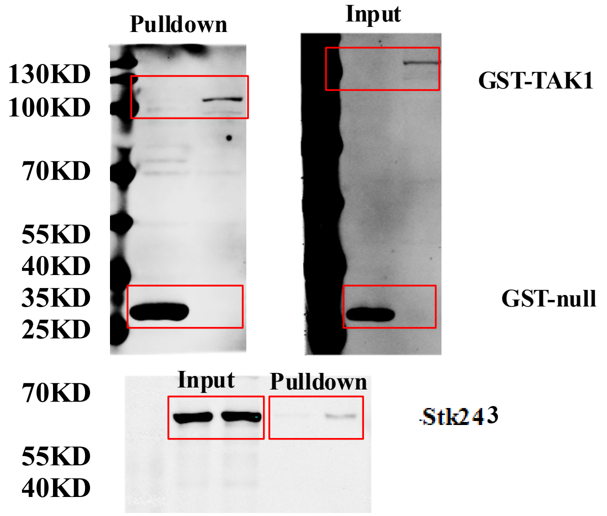

Fig6D

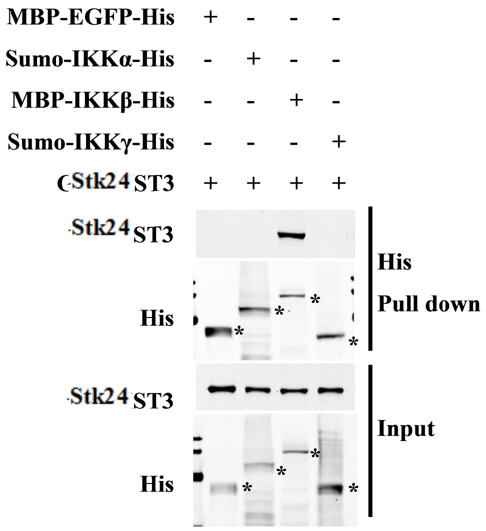

Full gel for Fig6D

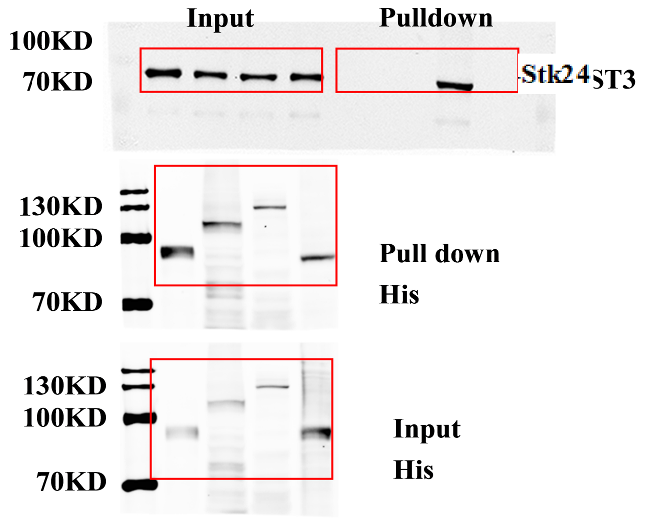

Fig6E

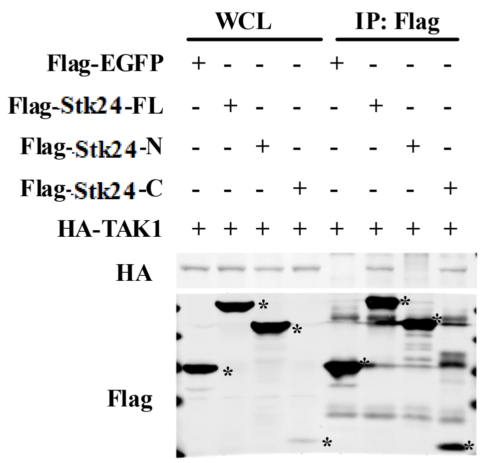

Full gel for Fig6E

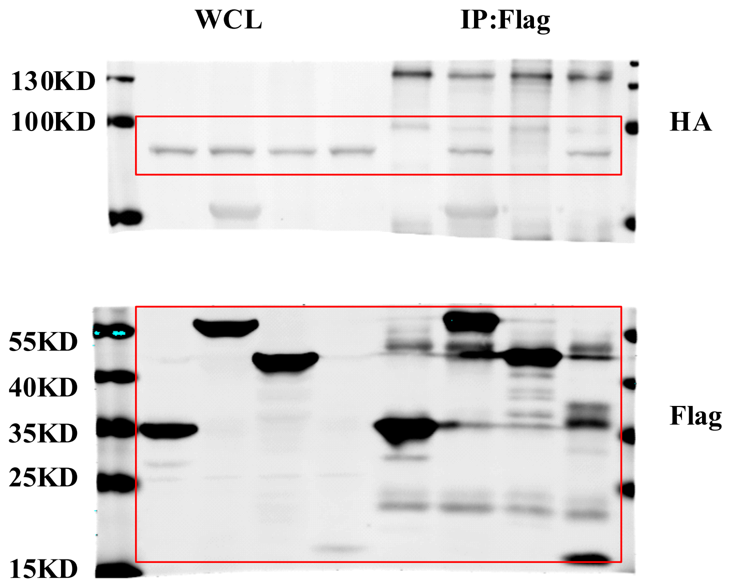

Fig6F

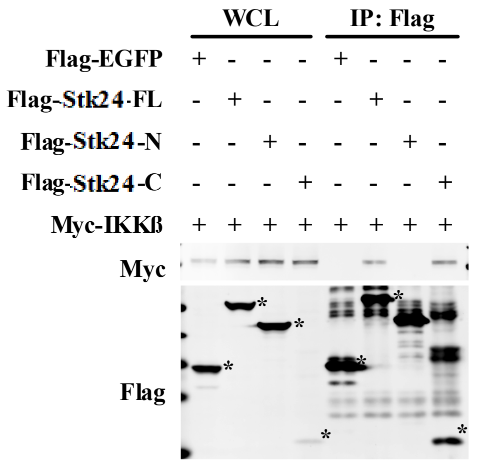

Full gel for Fig6F

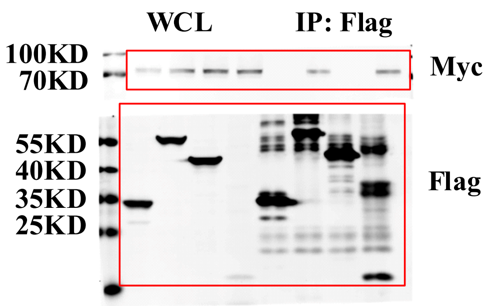

Fig6G

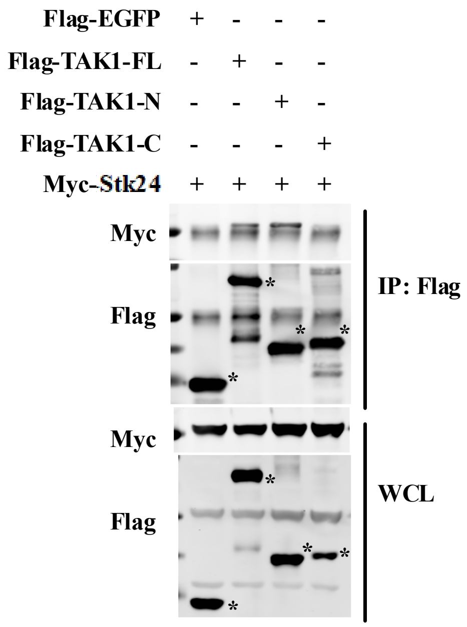

Full gel for Fig6G

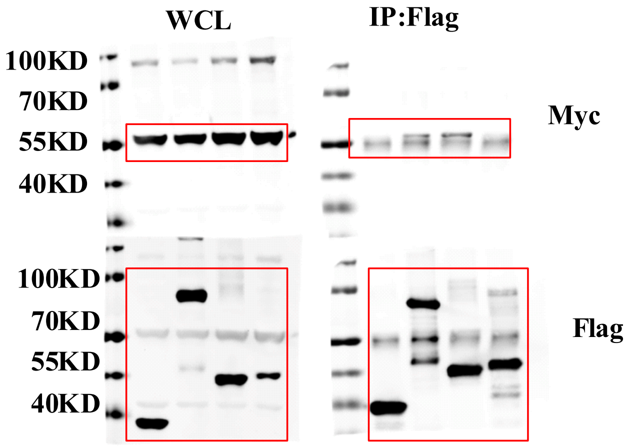

Fig6H

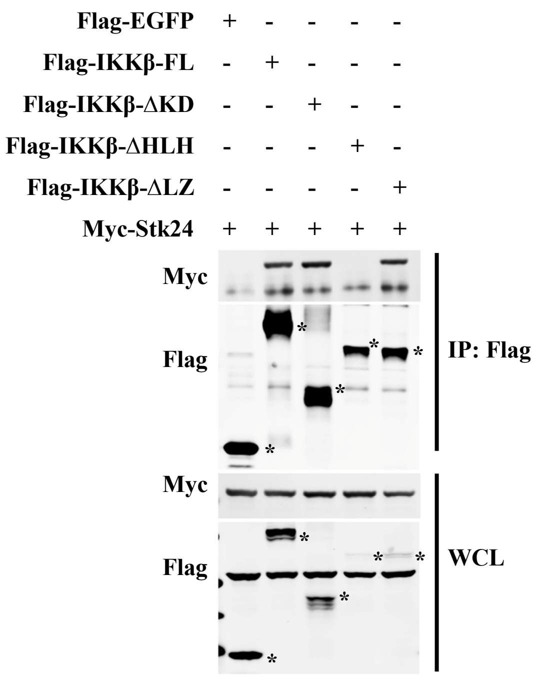

Full gel for Fig6H

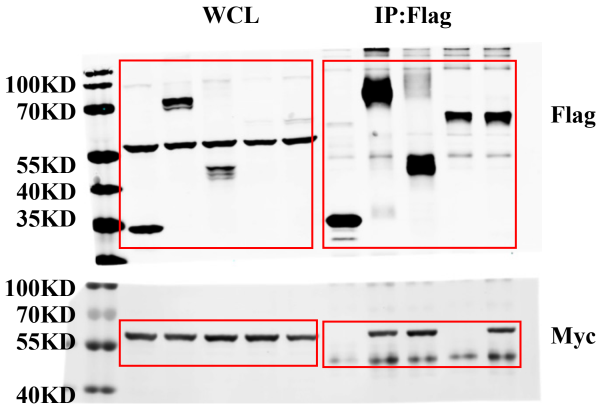

Fig6I

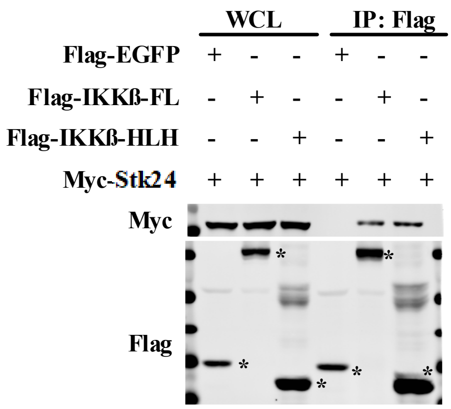

Full gel for Fig6I

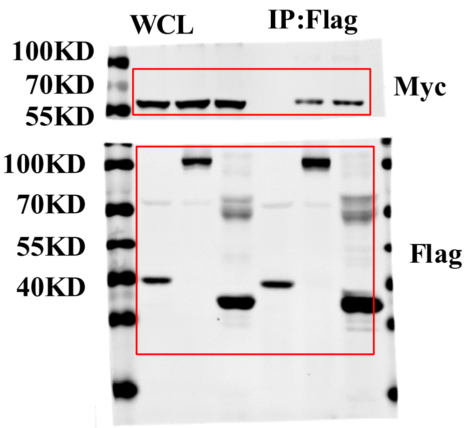

Fig7A

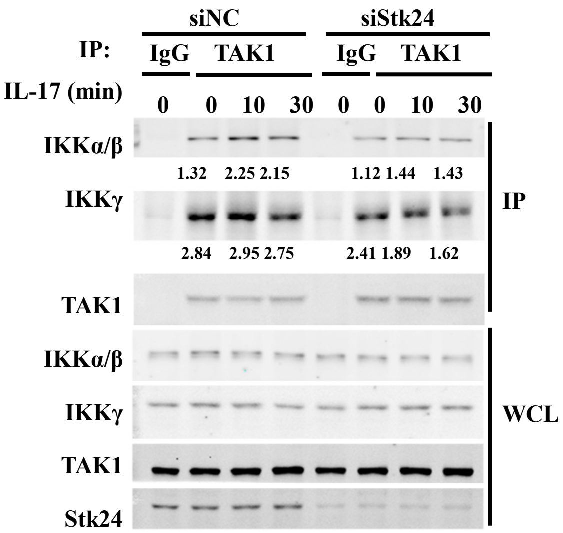

Full gel for Fig7A

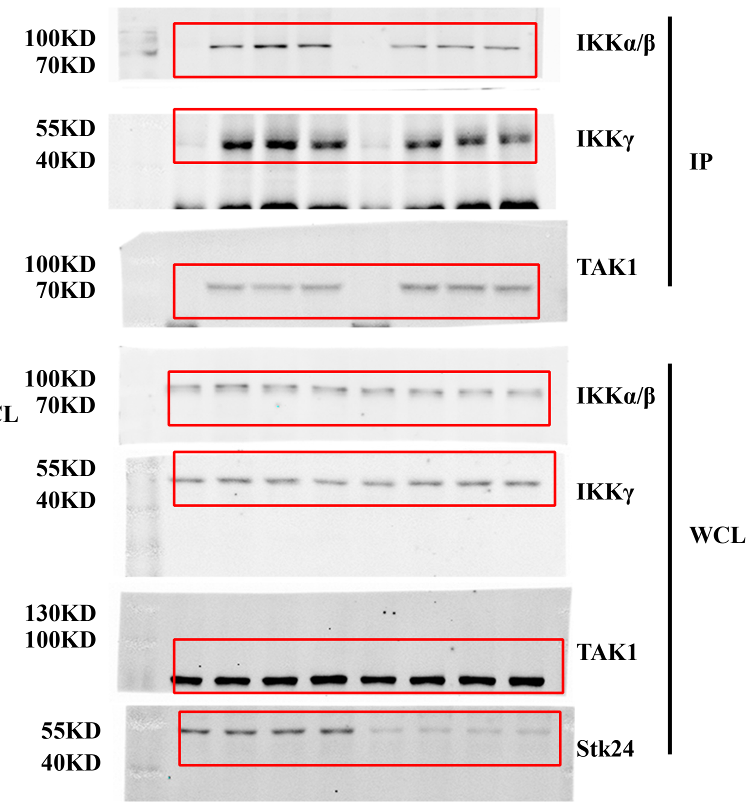

Fig7B

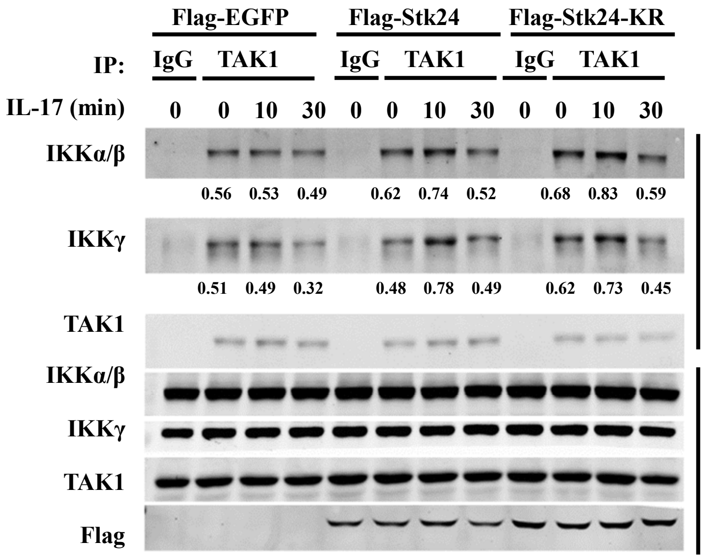

Full gel for Fig7B

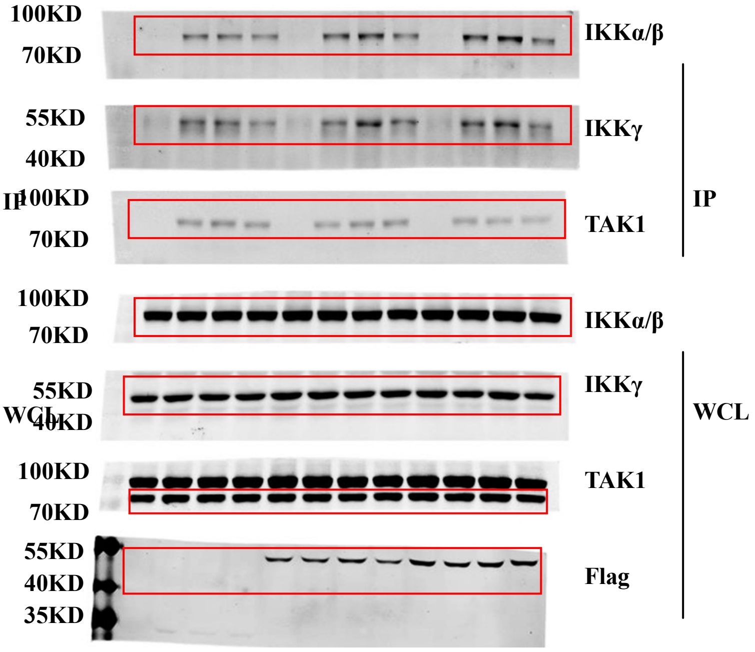

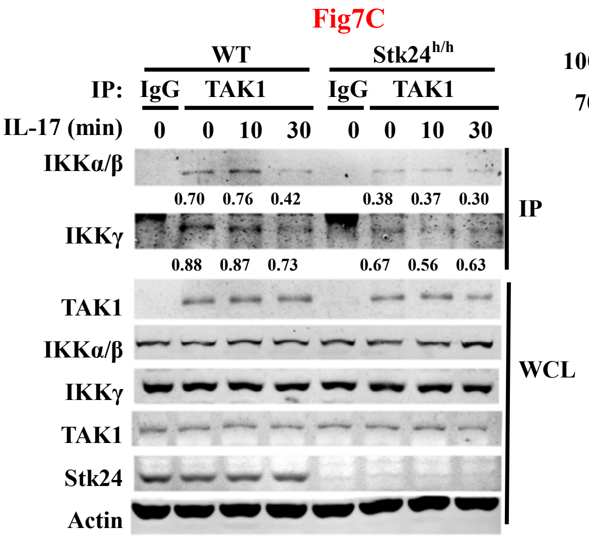
Fig7C
WT
Stk24h/h
IP:
IgG
TAK1
IgG
TAK1
IL-17 (min)
0 0 10 30 0 0 10 30
IKKα/β
0.70 0.76 0.42 0.38 0.37 0.30
IKKγ
0.88 0.87 0.73 0.67 0.56 0.63
IP
TAK1
IKKα/β
IKKγ
TAK1
Stk24
Actin
WCL

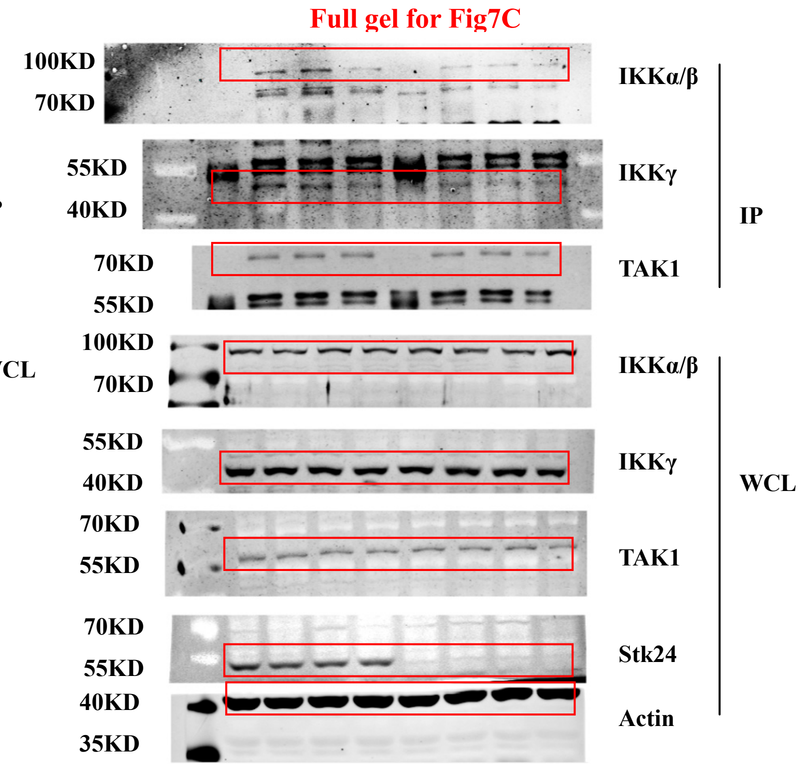
Full gel for Fig7C
100KD
70KD
IKKα/β
55KD
40KD
IKKγ
IP
70KD
55KD
TAK1
100KD
70KD
IKKα/β
55KD
40KD
IKKγ
WCL
70KD
55KD
TAK1
70KD
55KD
Stk24
40KD
35KD
Actin

FigS1B

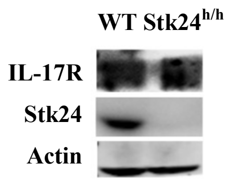

Full gel for FigS1B

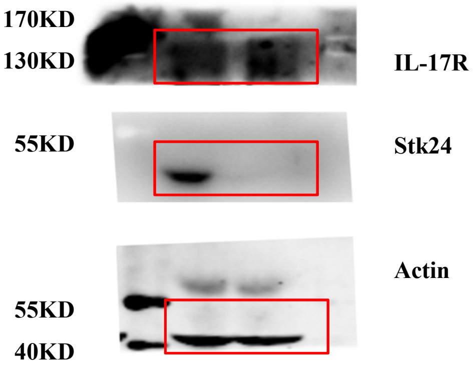

FigS2A

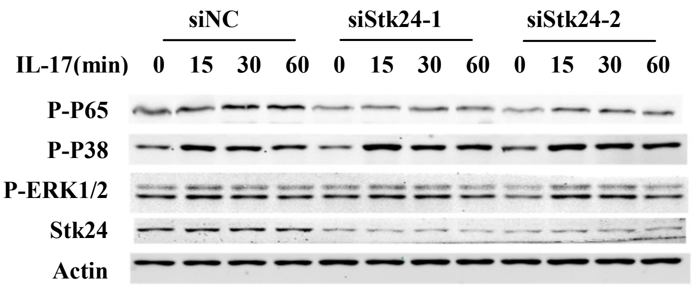

Full gel for FigS2A

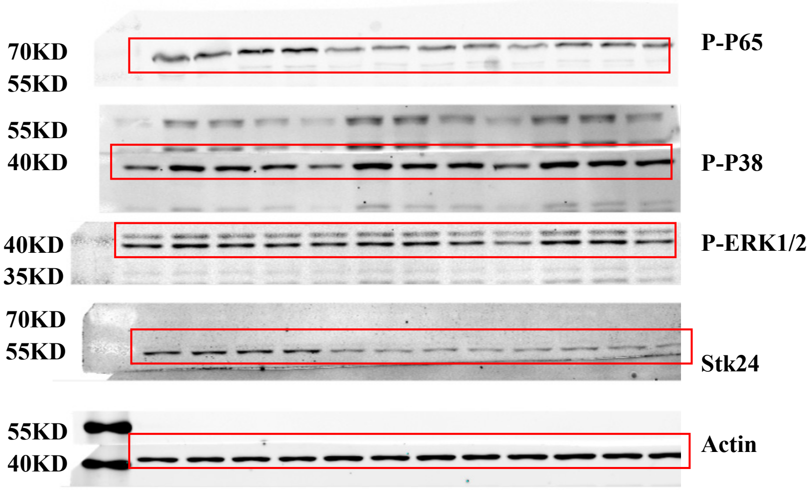

FigS2B

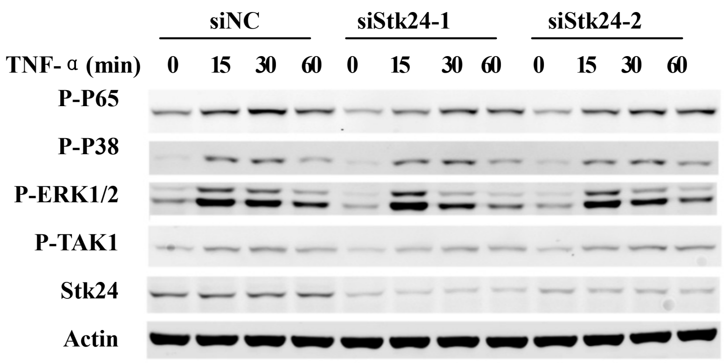

Full gel for FigS2B

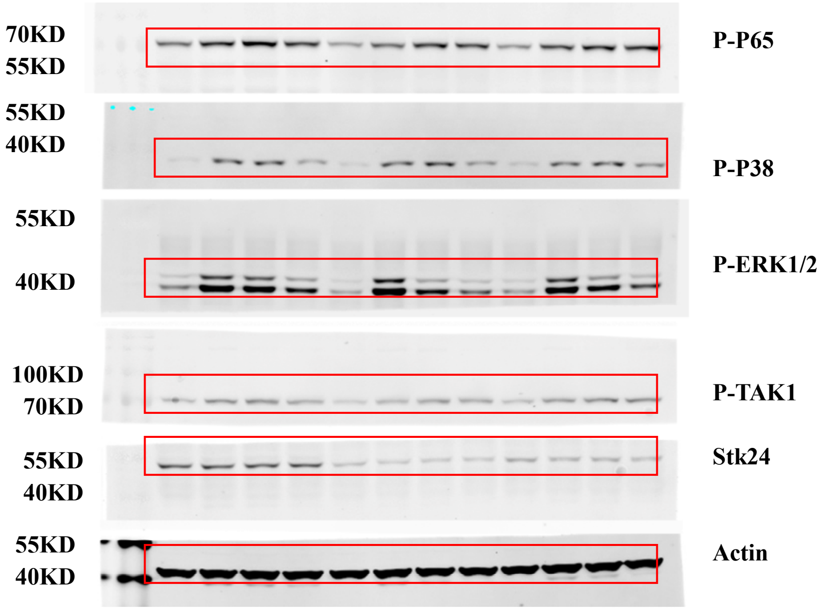

FigS2C

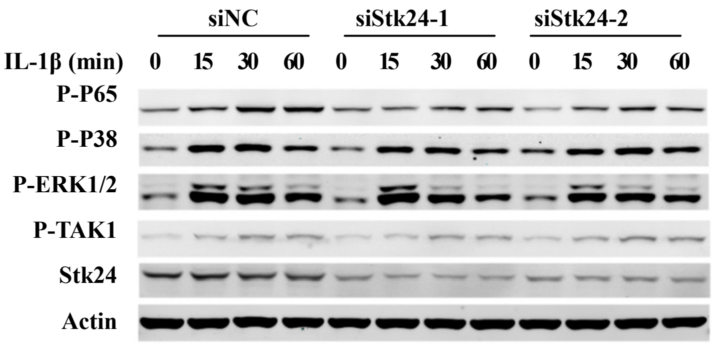

Full gel for FigS2C

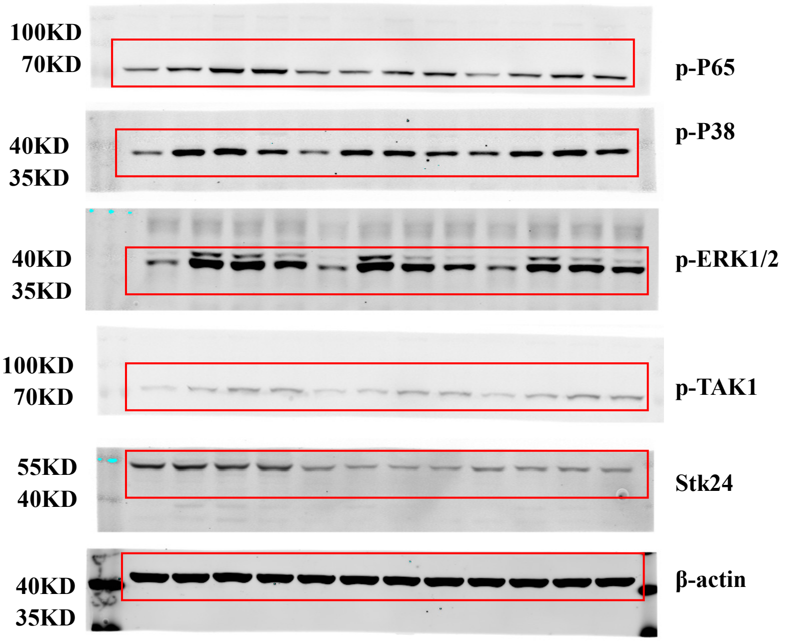

FigS2D

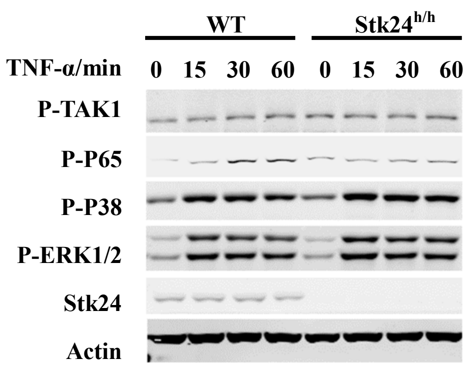

Full gel for FigS2D

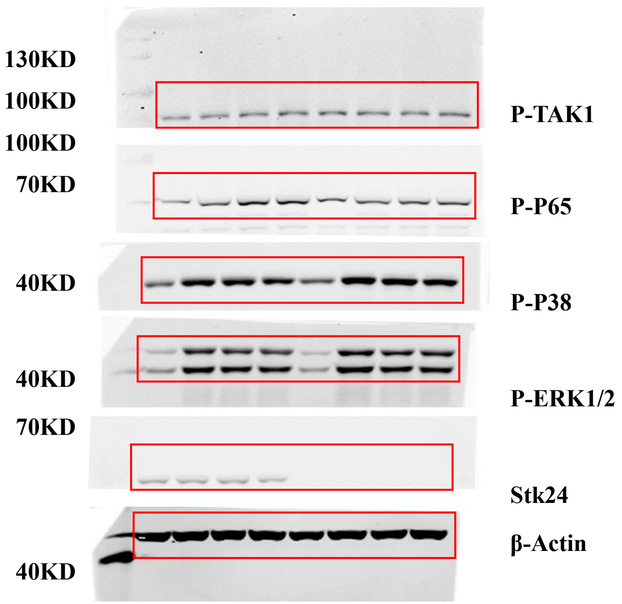

FigS2E

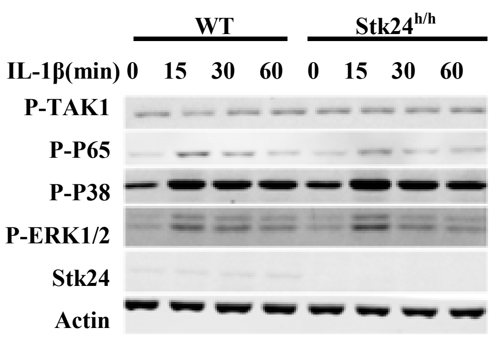

Full gel for FigS2E

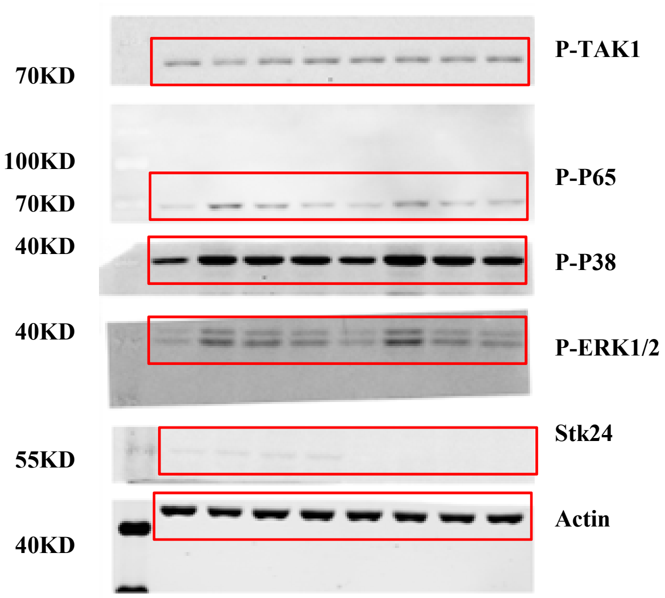

FigS4A

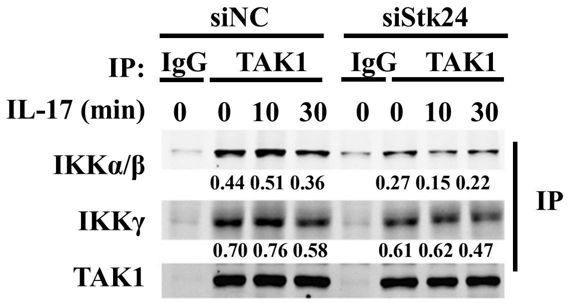

Full gel for FigS4A

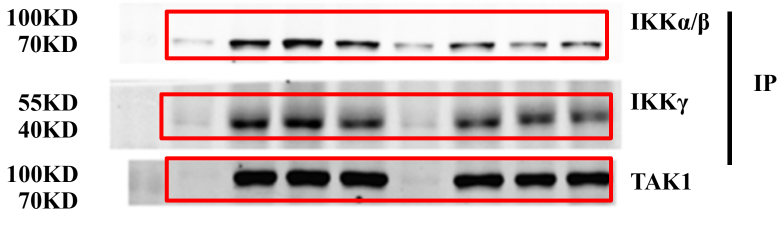

FigS4B

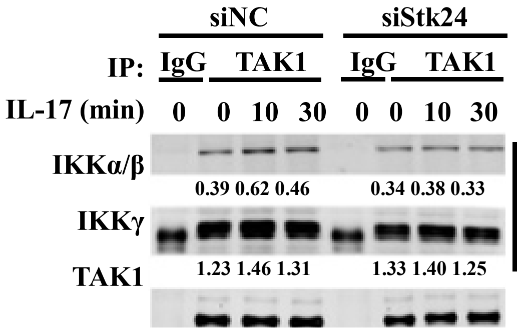

Full gel for FigS4B

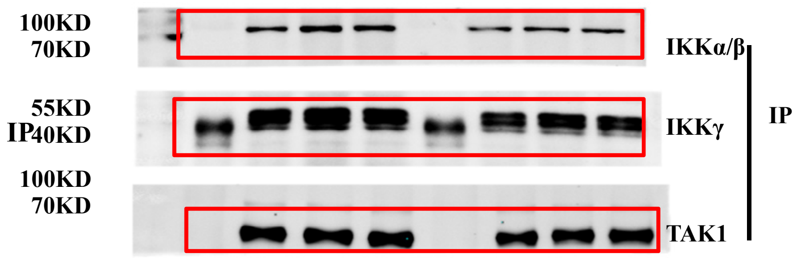

FigS4C

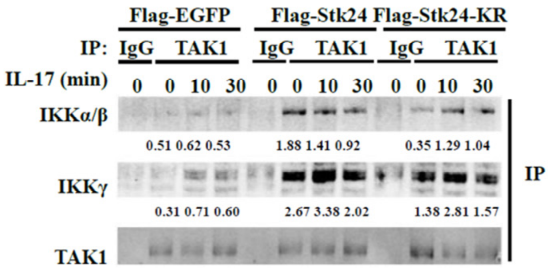

Full gel for FigS4C

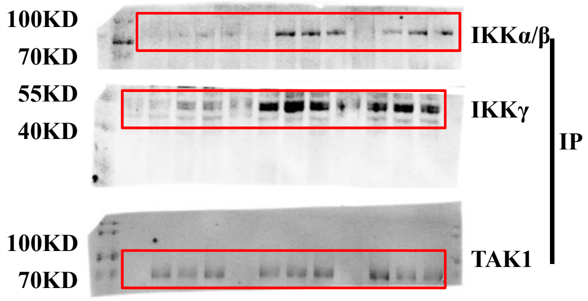

FigS4D

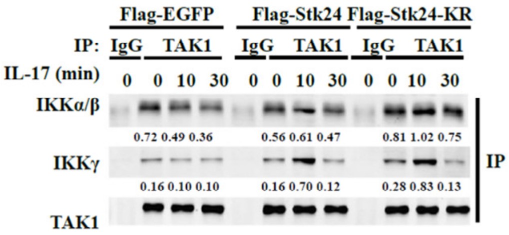

Full gel for FigS4D

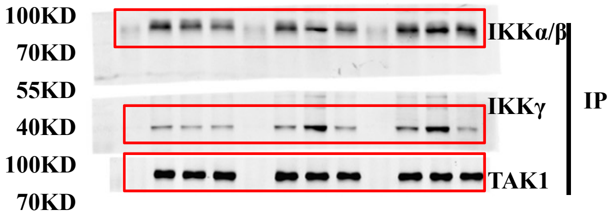

FigS4E

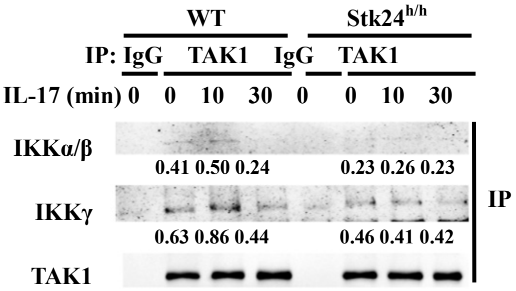

Full gel for FigS4E

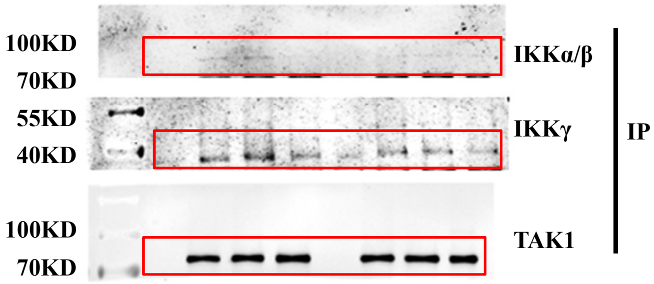

Supplement: Supplementary file 1 [file Data_Sheet_1.PDF]
